# High Sorption Efficiency of Purified Clinoptilolite-Tuff for Aflatoxins B1 and M1: A Case Study in Plant-Based Beverages and Milk

**DOI:** 10.3390/ijms262311265

**Published:** 2025-11-21

**Authors:** Carmen Ranftler, Magdalena Zehentner, Cornelius Tschegg, Dietmar Nagl

**Affiliations:** GLOCK Health, Science and Research GmbH, Hausfeldstrasse 17, 2232 Deutsch-Wagram, Austria; carmen.ranftler@glock.at (C.R.); magdalena.zehentner@glock.at (M.Z.)

**Keywords:** zeolite, purified clinoptilolite-tuff (PCT), aflatoxins, milk, plant-based beverages, gastrointestinal tract, adsorption, food intoxication, artificial fluids, ELISA (enzyme-linked immunosorbent assay)

## Abstract

Aflatoxins (Afs) belong to the most hazardous mycotoxins. Their detrimental effects on humans and higher animals are widely known, and actions are poised to avoid their synthesis already in the developing plant. However, this is often not effective enough or even not practicable, and hence, contaminated food and feed are consumed, resulting in severe health impairment. The use of adsorbents is one of the possibilities for the reduction or impediment of the venomous action after the intake of toxic food and feed. Purified clinoptilolite-tuff (PCT) was used as a binder of aflatoxins B1 and M1 in experiments with plant-based beverages as matrices for AfB1, while milk and dairy products of cow, sheep, and goat were the respective media for AfM1 contamination. Human gastrointestinal conditions were simulated by adequate temperature, movement, pH values, incubation times, and artificial juices. Analyses were implemented by appropriate ELISA assays for both toxins. PCT showed high affinity and kinetic velocity for AfB1 and AfM1. It neutralized irreversibly almost all toxins used with only traces detected after desorption experiments. PCT eliminated both plant-based beverages as well as milk and dairy products efficiently in a dose-dependent manner. This may offer a powerful method for minimizing the health risks of unavoidable aflatoxin exposure.

## 1. Introduction

### 1.1. Mycotoxins with a Special Focus on Aflatoxins

The ubiquitous filamentous fungi (molds) can produce venomous secondary metabolites, so-called mycotoxins, consisting of highly diverse organic structures with several heteroatom-containing groups with a molecular weight of 0.3–0.7 kDa [1]. Contrary to bacterial toxins, mycotoxins are not composed of proteins. They are dose-related toxic for higher animals, when incorporated by food and feed [2]. Although mycotoxins are mostly found in traces, even low concentrations can have severe effects due to their high toxicity potential—resulting in carcinogenic (liver, breast, lung, gallbladder, esophageal), hepatotoxic, teratogenic, immunosuppressive, genotoxic, mutagenic, nephrotoxic, cytotoxic, neurotoxic, embryotoxic, gastrointestinal and pulmonary toxic effects, dysfunction of the reproductive system, and estrogenic impact in both animals and humans [3,4,5,6]. Mycotoxicosis can be acute or chronic, depending on the duration of exposure to the mold toxins. There is a connection between climate and mycotoxin contamination: the warmer, the higher the mycotoxin levels are found [3]. In Europe, the reports of aflatoxin infestations are escalating as the temperature peaks are rising and the weather conditions are getting more extreme [7].

So far, more than 250 mold species have been discovered, which produce a total of more than 400 different mycotoxins. The Commission of the European Union regulates in 2023/915 [8] “On maximum levels for certain contaminants in food and repealing Regulation (EC) No 1881/2006” [9] the amount of several mycotoxins, as they are aflatoxins, ochratoxin A, patulin, deoxynivalenol, zearalenone, fumonisins, citrinin, ergot sclerotia, and ergot alkaloids. Multi-toxin occurrence is possible as most molds can synthesize several mycotoxins simultaneously, and more than one type of mold may infest food and feed. The appearance of a couple of mycotoxins can lead to synergistic, additive, or antagonistic effects for the affected organism, depending on their combination [3,4,10]. Moreover, there is ongoing research in the interaction of aflatoxins with other contaminants like pesticides, heavy metals, algal toxins, and polychlorinated biphenyls [4].

Aflatoxins (Af) are the most hazardous mycotoxins for humans and animals [3]. They were discovered in the early 1960s as the cause of a massive death of animals starting in the late 1950s, termed “turkey X disease”. The source of poison killing a total of 100,000 turkeys on a poultry farm in London was aflatoxin-contaminated groundnut meal from Brazil [4].

Members of the *Aspergillus* genus produce these highly toxic secondary metabolites, of which a total of more than 20 different representatives are known. Their toxicity varies, and six of those are the most significant ones: aflatoxins B1, B2, G1, G2, M1, and M2; with AfB1 being the most toxic one for humans and animals. The following *Aspergillus* species are known as toxin producers: *A. flavus*—the name giver of this mycotoxin class, aflatoxins, discovered in 1961, *A. parasiticus*, and *A. nomius*—all three belonging to the section *Flavi*. By 2025, already 28 aflatoxin-producing species from the *Aspergillus* sections *Flavi*, *Nidulantes*, and *Ochraceorosei* are known [4,11,12,13,14].

Aflatoxins, as furanocoumarin derivatives with slightly acidic character, are very durable molecules as they persist throughout food processing and maintain both their chemical and thermal stability (degradation only at >240 °C)—hence, being not only detectable in the final consumable product but also being still highly toxic for humans and animals [6]. The International Agency for Research on Cancer (IARC) classified naturally occurring mixtures of aflatoxins (AfB1, AfB2, AfG1, and AfG2) as group 1 carcinogens for humans in 1987 [4,6,11].

Mycotoxins lead to economic losses agriculturally—according to the Food and Agriculture Organization of the United Nations (FAO), >25% of the agricultural production is mycotoxin-contaminated—and in meat and milk production, so that several strategies have been already developed to prevent the synthesis of those from pre-harvest to post-harvest, during storage, and production [3,11,15], or to detoxify goods at least partially by physical, chemical, and biological methods, when already found in food and feed [4,5]. Currently, it is estimated that half of the human population is chronically exposed to aflatoxins via contaminated food [4,7]. There are reports that aflatoxin carry-over was found in milk, porcine tissue, and eggs. Aflatoxins enter the body not only by ingestion, but also by inhalation and dermal contact [7].

As early as 1965, the US Food and Drug Agency (FDA) released an initial regulation on the total amount of aflatoxins in foods, defining 30 µg/kg as the maximum level, lowering it to 20 µg/kg in 1966 [4]. Nowadays, in the European Union, the maximum level of aflatoxins in various raw materials and foodstuffs is regulated by the Commission Regulation (EU) 2023/915, depending on the commodity and its manufacture for children and adults. This refers to aflatoxin B1 alone as well as the total concentration of aflatoxins B1, B2, G1, and G2. However, there is no current legislation for plant-based beverages [6].

#### 1.1.1. Aflatoxin B1-Contamination of Plant-Based Beverages

The consumption of animal milk has been declining for years in Europe and the United States of America since the mid-1940s, and alternatives in the form of plant-based beverages were introduced on the market [16]. Among those products, soy-based ones have been established as a cow milk substitute in infant formula in the USA since 1917 [16]. Also, other plant-based drinks have been invented and consumed homemade for centuries before being available commercially [16]. Today, almond drinks are the most prominent plant-based beverages on the market [16].

The increase in consumption of plant-based beverages is based on several reasons, as there are lactose intolerance, milk protein allergies, and hypercholesterolemia, vegetarianism and veganism, animal welfare, lower environmental impact, and health awareness [6,16,17,18].

Plant-based beverages can be categorized into different subgroups depending on their source, as is depicted in Table 1 for widely available examples of non-dairy drinks.

To cover the broad spectrum of plant-based beverages, this study examined drinks made from almond, hazelnut, soy, oat, and cashew. These products were purchased from various supermarkets in Austria, where they are widely available.

Already, the raw materials themselves used for plant-based beverages are often contaminated by different kinds of molds, transferring them during the processing to the final drink. Aflatoxin B1 is classified as a carcinogenic and genotoxic substance, and hence, there is no safe threshold for the carcinogenic effect established. Children (aged 0.5 to <6 years) are the most vulnerable group concerning mycotoxin intoxication, as in relation to their bodyweight, they consume larger amounts of food than adults. In a small study conducted in Germany, in 23 out of 24 samples of almond drink, aflatoxins were detected. When assuming children consumed these drinks as an alternative to cow milk and their amount corresponded to that of cow milk usually consumed in this age category, the assessment of health risk was a medium likelihood for health impairments [20].

Plant-based beverages are based on water extracts of dissolved and disintegrated plant material. They feature a complex matrix composed of proteins, carbohydrates, lipids, and fibers [19]. To differentiate the sources of plant-based and animal-derived beverages, the European Union defined clear labeling in the Common Organisation of Markets Regulation (EU) No 1308/2013 [21] that the term “milk” was strictly reserved for animal descent [18].

#### 1.1.2. Aflatoxin M1-Contamination of Animal Milk and Dairy Products

Milk and milk products are an important source of various essential nutrients (calcium, phosphorus, essential amino acids, and vitamins) and are recommended in many dietary health guidelines. Dairy products contribute worldwide 49% of dietary calcium, 15% of dietary fat, and 12% of dietary proteins [18,22].

Aflatoxin B1, the prevalent and most toxic aflatoxin [7], is ingested into the animal organism via mold-contaminated feed, then adsorbed and further processed by the cytochrome P450 system in the liver to a final rate of 0.3–6.2% AfM1. Thus, there is a direct relation between the AfB1 levels in contaminated food and feed and the AfM1 levels finally found in animal and human milk. After 12–24 h post-food intake, AfM1 is excreted into the milk of the lactating mammal. Its level diminishes after 72 h if the ingestion of contaminated food was just a single event. AfM1 binds to casein, which contributes to circa 80% of the total protein content in milk. Milk, cheese, and yogurt derived from aflatoxin-exposed animals occasionally possess contents of AfM1. As aflatoxins are embryotoxic at high concentrations and have the ability to strongly influence the health of newborns and children (stunting, underweight, neurological impairment, immunosuppression, mortality), studies around the world (especially with tropical climate and pronounced drought of extended periods, which leads to an increased production of mycotoxins) are performed to identify the aflatoxin M1 amounts found in human milk after consumption of AfB1 contaminated plant products or AfM1 containing dairy goods. A direct correlation of higher concentrations of AfM1 in milk and dairy products and elevated levels of AfM1 in human milk samples could be established [2,7,22,23]. Besides AfM1, representing 95% of aflatoxins detected in milk, there are traces of other metabolites, namely AfM2, aflatoxicol, AfM4, and AfQ1 [7].

For this study, various milk products (buttermilk, whey, fat-reduced, organic, and whole milk) derived from three different species (cow, sheep, goat) were used.

Aflatoxin M1 and AfM2 are the monohydroxylated derivatives of AfB1 and AfB2. Due to their very heat-robust structure, AfM toxins cannot be removed by pasteurization or thermal treatment, and it is impossible to completely purify raw milk from them. AfM1, the “milk toxin”, is classified by the IARC as a 2B carcinogen for damaging DNA and may cause various cancer types [24]. Aflatoxin M1 cannot only be found in milk, but also in urine, kidney, and liver [4]. Moreover, AfM1 was also documented in plants being synthesized by *Aspergillus* spp. through a different synthetic pathway, either without prior generation of AfB1 or by the metabolism of insect pests [7]. By 1977, the US FDA limited the AfM1 level in milk to 0.5 µg/kg—a value still valid today [4]. The European Union has established the ALARA (As Low As Reasonable Achievable) system, which sets the AfM1 maximum permissible level in milk to 0.05 µg/kg. To hinder AfM1 from carry-over, the maximum residue level of AfB1 in feed for lactating cows was set in the European Union to 5 µg/kg and in the United States of America to 20 µg/kg. Other countries also defined maximum residue levels for AfM1, which are inconsistent around the globus. In some countries, regulation limits vary as they are adjusted to the mycotoxin contents [25,26]. Finally, there are still many states without regulation at all, mainly in the developing nations [7,24].

AfM1, like all aflatoxins, can cause acute and chronic aflatoxicosis. Even small amounts are concerning with long-term exposure. In developing countries, where little to no detection, monitoring, and regulation is exercised, outbreaks of acute aflatoxicosis after consuming highly contaminated food or feed are reported. The carcinogenic effect of AfM1 was demonstrated to be one order of magnitude lower than that of AfB1 [7].

#### 1.1.3. Aflatoxin Decontamination

Prevention of aflatoxin synthesis is the optimal way to gain safe food and feed. However, it is often not possible to ensure aflatoxin-free foodstuff, and hence elimination or at least mitigation of aflatoxins is the practiced way to obtain consumable items. There are various invented physical, chemical, and biological methods to generate food and feed under the legally defined toxicological limit. Some are very rudimentary, others, though extremely technically challenging, and only applicable to certain specific commodities. Furthermore, their reducing efficiency is of different quality. While all methods aim for detoxification without altering the original material, this outcome is not always guaranteed. A prospective goal is to gain general non-toxic all-purpose decontamination methods that can be employed for a broad spectrum of food matrices, which do not influence the food and feed contents as well as their sensory characteristics [3,4,5]. Table 2 gives an overview of several of those interventions.

Different kinds of adsorbers were tested for their potential to bind mycotoxins. In the literature, examinations with nanoparticles, nanocomposites, and magnetic-activated carbon, but also with microfibers of plant origin (cereals and legumes) containing mainly cellulose, hemicellulose, and lignin, are described. Moreover, synthetic polymers (e.g., cholestyramine, divinylbenzene-styrene, and polyvinylpyrrolidone) proved to be effective mycotoxin binders [5]. Natural and modified aflatoxin adsorbers—amongst others described above—are activated carbon, calcium bentonite, diatomite, esterified glucomannan, hydrated sodium calcium aluminosilicate (HSCAS, marketed as NovaSil^®^), vermiculite, nontronite, montmorillonite, sepiolite, and zeolite [5]. The reduction or even elimination of mycotoxins in feedstuff for livestock has been practiced with natural adsorbents since the 1960s for decades now [27,28,29]. Thus, hazardous phytochemicals are bound before entering the organism via the gastrointestinal tract [3]. They can be added to food and feed or taken separately during meals to prevent aflatoxicosis [7].

Phyllosilicates (sheet silicates) belong to the broader group of aluminosilicates and are structurally distinct from tectosilicates (framework silicates); they consist of interconnected tetrahedral (Si^4+^, Al^3+^) and octahedral (Al^3+^, Fe^3+^, Fe^2+^, Mg^2+^) sheets, with both containing oxygen and hydroxyl groups [30]. Clay materials are composed primarily of such layered silicate minerals. For example, kaolin as well as bentonite, which is a composite material consisting of several minerals, are widely used for detoxification of milk [31,32].

Another subgroup of aluminosilicates is tectosilicates, in which each central silicon atom is coordinated by four oxygen atoms that link to adjacent tetrahedral units, forming an infinite structure. Zeolites belong to the tectosilicate group. They are predestined to bind polar molecules. This property is due to their large surface area, their net negative charge, and the high cation exchange capacity. One of the most abundant natural zeolites is clinoptilolite, with broad application as a feed additive, in water and air treatment, for nuclear waste management, as well as in agriculture and biochemical utilization [5,30].

### 1.2. Zeolite—Structure, Application, and Purification

#### 1.2.1. The Application of Zeolites as Sorbents for Various Substances

Geophagy, the intentional consumption of natural rock- or soil-derived substances as sorbents in the gastrointestinal tract, is a phenomenon described in animals and humans across centuries and all over the world [2]. Besides various types of clays, one group of such natural sorbents is zeolites, minerals known for their high sorption and ion exchange capacity [33]. Today, over 40 natural zeolites and more than 160 synthetic types are described, each tailored and utilized for specific applications based on their molecular affinity [28,29,34]. Zeolites are used for purification and modification of gases, (waste-)water treatment, binding of radioactive ions like strontium and cesium, and they function as soil amendments/conditioners/fertilizers and animal feed. Other application areas can be found in industry as building materials, soil stabilizers, and as part of special cements, and in the rubber/filler production, etc. For many years, natural zeolites have been used for medical purposes, in dietary supplements and cosmetics [28].

Their versatile properties are attributed to their overall net negative charge and the possibility of exchanging crystal-lattice ions with the environment without destroying their structure [29,33]. Aflatoxins are characterized by a positive charge, making them suitable candidates for adsorption by such alumosilicates [35]. They belong to molecules with higher polarity and are predestinated for binding to mineral adsorbers (>90% sorption), while other mycotoxins with lower polarity (like zearalenone, ochratoxin A, T2 toxin) have distinct lower adsorption rates (30–50%) [36].

Primary research on aflatoxin adsorption began with phyllosilicates. In 1978, Masimango et al. demonstrated that various clays could adsorb AfB1 from contaminated media with removal rates ranging from 70% to undetectable levels, and most clays bound AfB1 irreversibly [37]. In 1982, Applebaum and Marth showed that bentonite could eliminate up to 89% of AfB1 from naturally contaminated milk derived from cows administered with AfB1 in feed [38].

Natural zeolites, though chemically similar to phyllosilicates, differ in their crystal structure, forming a porous network of SiO_4_ tetrahedra. Partial substitution of Si^4+^ by Al^3+^ creates a negatively charged lattice that attracts and incorporates positively charged ions, while larger ions are adsorbed onto the surface. This structure underlies the high sorption capacity of zeolites [39,40], making their adsorption potential comparable to that of phyllosilicates. Clinoptilolite is one such natural zeolite.

#### 1.2.2. Clinoptilolite and Purified Clinoptilolite-Tuff (PCT)

Clinoptilolite is a mineral of the zeolite group, precisely of the heulandite type, being an individual mineral since 1960 [41]. It is the main mineral of clinoptilolite-tuff, which is found and mined globally, however, in varying amounts and quality. As formed in different geological eras by volcanic eruption and following mineralization reactions of glassy ash particles and saline alkaline lake or soil water, its mineralogical composition varies [28,39,41]. Clinoptilolite’s adsorption potential is restricted to molecules with maximum kinetic diameters of 3.5/3.8 Å [34]. There is a wide span of diameter indications published for aflatoxin molecules—for example, one can find values of 1–100 nm [42], 100 nm [43], and <1 µm [44]. Despite their broad pore structure, aflatoxins are too large to be absorbed by clinoptilolite; thus, only surface adsorption is possible. The raw material used in the presented experiments was sourced from a high-grade open-pit mine located in Nižný Hrabovec, eastern Slovak Republic [39]. This deposit derives from the Miocene and has been geologically investigated since 1974. It bears a clinoptilolite of the potassium-calcium type with low content of iron, magnesium, and sodium ions ((Ca_1.51_K_1.39_Mg_0.37_Na_0.15_)[Al_5.64_Si_26.36_O_72_]×11.77 H_2_O) [40,41,45]. Clinoptilolite (the only present zeolite mineral-phase) is accompanied in this tuff by other minerals like feldspar, plagioclases, cristobalite, and mica [45]. However, due to its volcano-sedimentary origin, clinoptilolite contains elements that are undesirable for prolonged human consumption. Those are eliminated by a fully quality-controlled and patented process involving ion exchange, micronization, and terminal heating. The purification process begins with crushing the raw material to a particle size of 0.5–4 mm. The crushed zeolitic material is then subjected to a sequence of ion-exchange treatments. Initially, it is flushed with a series of diluted acidic ammonium chloride (NH_4_Cl) solutions, followed by treatment with calcium chloride (CaCl_2_) solutions. Subsequently, the material is thoroughly washed with water (*aqua purificata*) and dried. In the final stage, the ion-exchanged and purified material is micronized using a jet mill and then thermally treated at 160 °C for 120 min. The resulting purified clinoptilolite-tuff (PCT) is safe for consumption and marketed in the USA as PCT or G-PUR^®^ [39,40,46].

**Figure 1:**
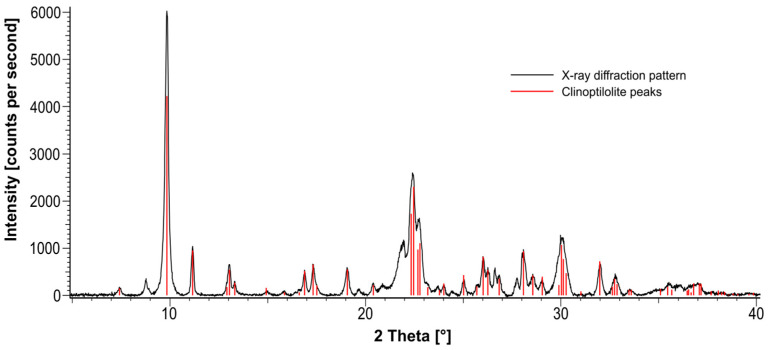
X-ray diffraction analysis of the PCT (batch-number 025-01-08-7-1-0-P-0-A) used for the experiments presented in this paper. In black, the diffractogram of the sample is drawn, while red marks correspond to the clinoptilolite peaks within the sample.

Formation history of the raw material in the open-pit mine in Nižný Hrabovec, as well as the comprehensive physico-chemical and mineralogical composition of the raw material and PCT, was intensively studied by Tschegg et al. [39,40] as well as Haemmerle et al. [47]. X-ray diffraction (XRD) data, particle-size distribution, and ion-exchange capacity of the batch used for performing the experiments of this paper are presented in Figure 1 and Table 3.

In prior experiments, this special purified form of clinoptilolite tuff (PCT) gave excellent results when applied as a binder of various organic compounds like gluten [48] and peanut protein [49], *Clostridium difficile* toxins A and B [50], and viruses [51,52]. The question was raised whether this material is capable of binding aflatoxins B1 and M1 not only in general, but also within complex matrices such as milk and plant-based beverages, and particularly under in vitro digestion conditions. Although in vitro experiments were performed, close attention was paid to mimic human conditions in the gastrointestinal tract. Tests were executed at 37 °C, corresponding to human temperature, slowly rotating to simulate gastrointestinal movements, and exhibited retention times of at least 30 min to 4 h in accordance with solutions remaining in the stomach and gut. The highly acidity and the almost neutral milieu of the intestine were imitated with test solution (pH 1.5) and phosphate buffer (pH 6.8) at first, and finally with complex matrices as artificial digestion juices present. Purified aflatoxins B1 and M1 were used to contaminate plant-based beverages (almond, hazelnut, soy, oat, and cashew) as well as milks and dairy products of different species (cow, sheep, goat), respectively. PCT was applied in realistic conditions, as 2 g per day is recommended for human internal application.

The PCT saturation capacity for AfB1-spiked almond drink in highly acidic conditions corresponded to 1.75-fold (14 µg AfB1/mg PCT), in nearly neutral ones to 0.5-fold (5 µg AfB1/mg PCT) of the maximum permitted value as is in the EU for 1 kg of almonds contaminated with 8 µg AfB1. Moreover, in artificial gastric and intestinal juice, PCT in a concentration of 1 mg/mL could adsorb approximately half of the AfB1 (8 µg/kg almond drink) added, corresponding to 4 ng AfB1/mg PCT. Taken together, a daily dose of PCT could neutralize at least the maximum legally permitted level of AfB1 in 1 kg of almonds, as there is no defined limit for plant-based beverages. Further experiments were extended to other types of plant-based beverages with AfB1 in phosphate buffer pH 6.8. The results gained with hazelnut, soy, oat, and cashew were comparable to those with almond drink, leading to the assumption that PCT can also bind and neutralize aflatoxin B1 in these complex matrices.

After confirmation that PCT can bind AfB1 in various plant-based matrices in relevant amounts, AfM1—as its hydroxylated metabolite—was in special focus. Generally, once milk is contaminated and dairy products are produced therefrom, AfM1 cannot be removed completely; hence, there is still a risk for consumers. Especially when organisms are exposed to AfM1 constantly, even small amounts of toxin can lead to health impairments. Similar experiments as with AfB1 underwent previously—using test solution pH 1.5, phosphate buffer pH 6.8, and after all artificial digestion juices—were then performed with AfM1. A binding with high affinity and velocity (80% in the first minute) of AfM1 to PCT in cow milk was verified and desorption experiments proved a stable connection between them: When mimicking the changing pH values during digestion (pH 2.0, 5.5, and 8.0) in a very simple artificial model and incubation for 4 h totally, only minor traces of AfM1 could be found in the medium afterwards. Good adsorption results were again received in artificial gastric and intestinal juices. Lastly, different kinds of cow milk (whole, fat-reduced, organic) and dairy products (whey, buttermilk) as well as organic milk from sheep and goat were tested for AfM1 binding to PCT and compared with each other. Remarkable differences could be seen. Buttermilk stood out as an outlier, possibly due to its unique viscosity and composition. Summarizing all results gained, it was demonstrated that the severe toxic aflatoxins AfB1 and AfM1 can be at least partially neutralized by purified clinoptilolite-tuff.

These preliminary results from laboratory experiments could be the basis for clinical trials. Particularly in regions of the world where aflatoxin contamination of food is unavoidable, PCT could potentially help to mitigate health problems.

## 2. Results

### 2.1. Analysis of AfB1 Sorption in Plant-Based Beverages by PCT

Nowadays, the consumption of milk replacers is common due to a variety of reasons, such as dairy intolerance, vegan diets, or simply taste. As plant-derived products are prone to fungal infestation, thereby being contaminated with mycotoxins, the first aim of this study was to determine if PCT could bind AfB1 in plant matrices used for human consumption. For experiments, different products derived from various plants were used: almond, oat, hazelnut, cashew, and soya drinks served as examples of nondairy drinks. According to nature, AfB1 was used for spiking of the plant-based beverages, as AfM1 derives from animal metabolism of AfB1 and is of interest when regarding milk and milk products. AfM1 is not relevant in the production of plant-based beverages.

The primary objective of the first experiments was to saturate PCT with AfB1 to evaluate the maximum adsorption capacity of PCT for AfB1 at pH levels corresponding to physiological conditions.

#### 2.1.1. Saturation of PCT with AfB1 at Different pH Values Relevant to Human Digestion

Characterizing the sorption characteristics of PCT for AfB1, a main issue was to obtain an idea of the maximum adsorption capacity of PCT for AfB1 without any compounds that might interfere with the sorption reaction. For this purpose, liquids with increasing toxin concentrations were incubated without (controls) and with PCT at a fixed concentration of 1 mg/mL to achieve saturation of the sorbent with the toxin and thereby determine the maximum adsorption capacity of PCT for AfB1. Moreover, this low PCT concentration should avoid the need for excessively high AfB1 concentrations. The adsorption tests were carried out in the matrix of a test solution representing gastric acidity (pH 1.5) and an almost neutral phosphate buffer (pH 6.8) corresponding to intestinal pH. After incubation at 37 °C for 30 min (rotating at 17 rpm), the test samples were centrifuged to separate PCT-bound and free AfB1. The toxin in the appropriately diluted supernatants was quantified by ELISA.

The quantity of adsorbed toxin was calculated by difference and plotted against the respective concentrations of free toxin. The resulting isotherms were fitted using the established sorption models of Freundlich, Langmuir, and a combination of both, which is also called the Sips model.

**Figure 2:**
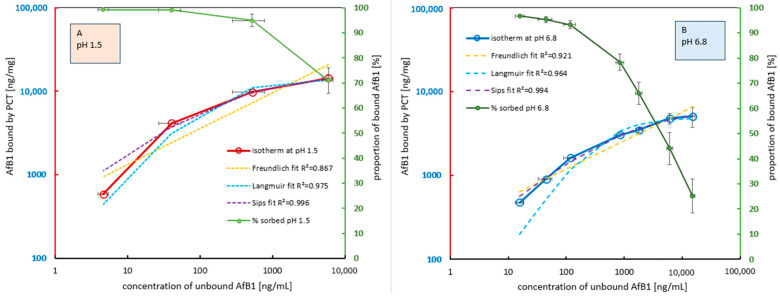
Adsorption of AfB1 by PCT in test solution at pH 1.5 (red curve, (**A**)) compared to that of phosphate buffer at pH 6.8 (blue curve, (**B**)). The isotherms reflect the amount of adsorbed AfB1 per mg PCT as functions of the concentration of free (unbound) toxin measured under equilibrium conditions. The x-axis and primary y-axis are logarithmically scaled to better represent low concentrations of free toxin under conditions where high amounts of toxin are bound. The proportion of bound toxin is displayed in the corresponding green curves, which are referenced against the linearly scaled secondary y-axis. Incubation of the AfB1 solutions without (controls) and with PCT at a constant concentration of 1 mg/mL was carried out for 30 min at 37 °C and 17 rpm rotation. After centrifugation to separate free from PCT-bound AfB1, the supernatants were appropriately diluted and analyzed by ELISA for free AfB1. Isotherms were calculated and fitted using commonly applied adsorption models (Freundlich, Langmuir, and Sips). The correlation coefficients (R^2^) of the respective approximation curves are indicated in the figure legend. Graph represents data including standard deviations of 2 individual experiments at each pH, conducted in quadruplets each.

Figure 2 illustrates the results of two consecutive experiments conducted at pH 1.5 and pH 6.8, respectively, each performed in quadruplicate, plotted within a double-logarithmic coordinate system. For improved clarity and interpretability, the sorption curves obtained at pH 1.5 (panel A, left) and pH 6.8 (panel B, right) are presented separately.

It is evident that the sorption characteristics differ significantly between pH 1.5 and pH 6.8, despite the comparable range of toxin concentrations. The higher position of the sorption isotherm at pH 1.5 (panel A, red curve), as well as the corresponding curve representing the relative proportion of bound toxin (panel A, green curve), indicates that under highly acidic conditions, a greater amount of toxin is retained by the same quantity of PCT compared to near-neutral pH 6.8. At the highest applied AfB1 concentration of approximately 20,000 ng/mL—which is far beyond levels realistically encountered under physiologically digestive conditions—approximately 70% of the toxin is still bound at pH 1.5. This corresponds to an adsorption capacity of roughly 14,000 ng AfB1 per mg of PCT. Under these conditions, saturation is only beginning to occur.

Compared to pH 6.8, and under otherwise identical conditions, only about 25% of the applied toxin is bound (panel B, green curve). This means that approximately 15,000 ng/mL of AfB1 remain free in the supernatant, while around 5000 ng of toxin per mg of PCT are retained (panel B, blue curve). Notably, sorption saturation occurs already at toxin concentrations below the maximum applied level.

Moreover, at all lower toxin concentrations, nearly all AfB1 is bound under acidic conditions (99–95%), whereas at pH 6.8, the proportion of adsorbed toxin progressively decreases with increasing toxin concentration to reach 45% at the initial AfB1 level of 10,000 ng/mL (green lines in Figure 2A,B).

Although a flattening of the curve was observed, a clear plateau of the adsorption curve was not yet evident (see Figure 2A, red line). Up to an initial toxin concentration of 4000 ng/mL, the majority of the toxin was effectively bound; at 10,000 ng/mL, almost 95% of AfB1 was still sorbed to PCT.

Regarding the evaluation of the data against various sorption models, the Freundlich model produced the lowest correlation coefficients. This outcome has been expected, as the Freundlich model does not assume saturation of the adsorbent, whereas the data clearly indicate a saturation effect. In contrast, the Langmuir approach shows both visually and computationally a much better fit with the empirically obtained data series. The Langmuir model is based on the concept of idealized monolayer adsorption and a finite number of discrete sorption sites on the adsorbent surface, which aligns well with the observed saturation behavior. The best approximation is achieved using the combined Langmuir/Freundlich model, also known as the Sips model, which produces near-ideal correlation coefficients just below 1 in the present case for both pH conditions. According to the Langmuir equation, the maximum adsorption capacity is calculated to be approximately 14,000 ng/mg at pH 1.5 and just below 5000 ng/mg at pH 6.8. Based on the Sips model, the calculated maximum adsorption capacities are approximately 16,000 ng/mg for pH 1.5 and 6200 ng/mg for pH 6.8.

#### 2.1.2. Sorption of AfB1 by PCT in Spiked Almond Drink During Artificial Digestion in Synthetic Gastric and Intestinal Juice

Aflatoxin B1 is a well-known contaminant of almonds and hence of all products derived from this stone fruit. For this reason, it was chosen as the first representative of plant-based beverages to be analyzed in experiments deliberately contaminated with a distinct concentration of AfB1. Artificial gastric and intestinal fluids served as matrices, wherein PCT was supplemented for AfB1 sorption. Again, analyses were conducted using the ELISA technique. To prevent the antibodies from enzymatic activity, the enzymes pepsin and pancreatin were deactivated thermally prior to the mixing of the artificial fluids.

**Figure 3:**
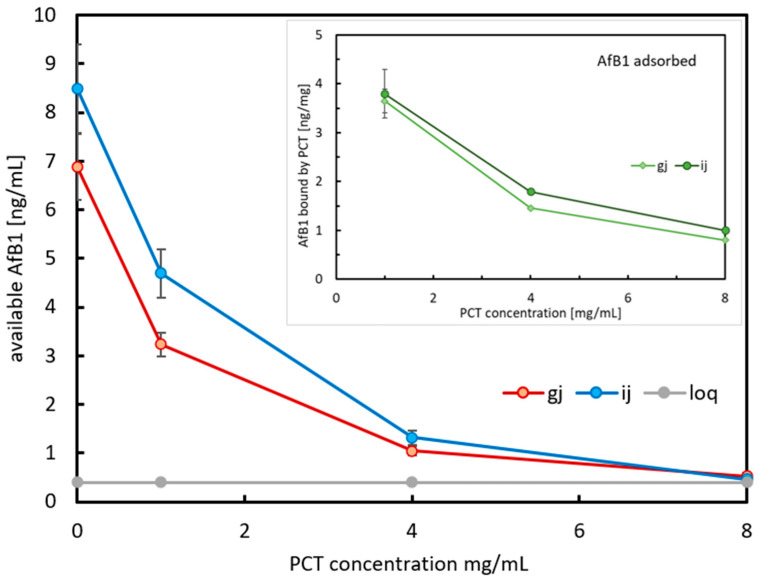
Adsorption of AfB1 in spiked almond drink by PCT in artificial gastric and intestinal juice. The incubation took place rotating at 17 rpm for 30 min at 37 °C without (control, starting point) and with PCT at different concentrations (1 mg/mL, 4 mg/mL, and 8 mg/mL). After centrifugation to separate free from PCT-bound AfB1, the supernatants were analyzed by ELISA for the residual content of free AfB1. Artificial gastric and intestinal juices are depicted as red and light green or blue and dark green lines, respectively, while the limit of quantification (loq) is drawn in gray line. Data, including standard deviations of 2 separate experiments consisting of quadruplets each, are shown.

Figure 3 illustrates the amount of AfB1 neutralization during a 30 min incubation under simulation of physiological conditions (37 °C, gentle rotation at 17 rpm) in artificial juices with or without (controls) PCT added. For both juices, a concentration of AfB1 corresponding to 8 µg/L was desired. This particular level of AfB1 for spiking the almond drink was chosen as the regulation for contaminants in the European Union defines 8 µg/kg as the maximum value for placing an almond on the market [8]. In the case of intestinal juice, the ELISA analyses showed that this value was even exceeded (~8.5 ng/mL, blue line), while regarding the results gained from gastric juice, only 7 ng/mL (red line) were detected. The binding of toxin was in direct correlation to the amount of PCT added. For both artificial fluids, the neutralization of AfB1 was similar—1 mg of PCT could neutralize approximately half of the AfB1 added. After using 4 mg of PCT, approximately 80% of AfB1 were sorbed to PCT. As shown in the inset plot (light and dark green lines for gastric and intestinal juice, respectively), the calculated binding ranged from 1 ng to 4 ng of AfB1 per mg of PCT.

#### 2.1.3. Sorption of Aflatoxin B1 by PCT in Selected Types of Plant-Based Beverages (Almond, Hazelnut, Soy, Oat, and Cashew Drinks) in Phosphate Buffer at pH 6.8

**Figure 4:**
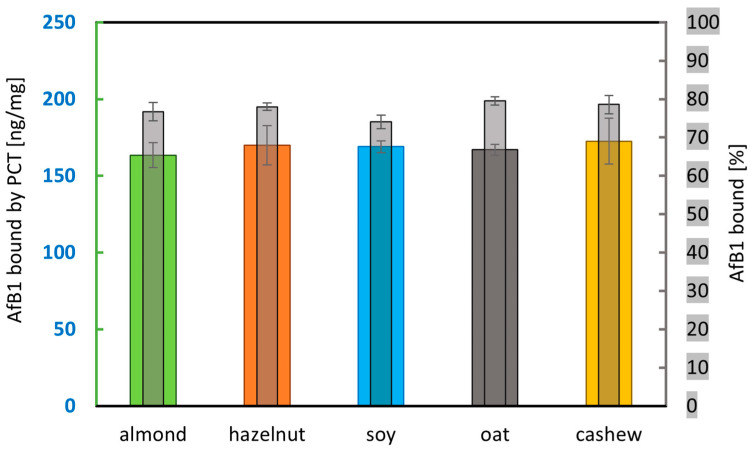
Adsorption of AfB1 by PCT tested in 5 different spiked plant-based beverages (almond, hazelnut, soy, oat, and cashew drinks) mixed with phosphate buffer pH 6.8 as a simple example of a part of the digestion process at intestinal pH. The initial toxin concentration of the experimental samples was set above 200 ng/mL. The incubation was carried out at 37 °C without (control) and with PCT at a concentration of 1 mg/mL for 30 min and 17 rpm. After centrifugation to separate the free from the PCT-bound AfB1, the supernatants were analyzed by ELISA for the residual content of free AfB1. The colored bars indicate the amount of bound AfB1 per 1 mg of PCT. The narrow gray bars show the relative proportion of bound toxin. The graph represents data including standard deviations of 2 individual experiments conducted in quadruplets each.

After successful sorption of AfB1 by PCT in spiked almond drink and artificial digestion fluids, the next step was to compare the sorption characteristics of PCT for AfB1 in various milk alternatives found—like almond drink—regularly on the market: hazelnut, soy, oat, and cashew drinks. Each tested product was spiked with a fixed high level of AfB1 above saturation conditions. Although the toxin concentration of about 200 ng/mL was unrealistically high, it was necessary to measure unbound AfB1 in the supernatants. The spiked plant-based beverages were mixed 1 + 4 (v + v) with phosphate buffer pH 6.8 to generate a simple model of artificial digestion, and with PCT suspension in a final concentration of 1 mg/mL. The used PCT concentration was decided to be low to achieve saturation of adsorbed and detection of AfB1 in the supernatants. Samples without PCT served as controls. Incubation was carried out as previously described to simulate roughly the human digestion process (30 min, 37 °C, rotating at 17 rpm). PCT and adhered AfB1 were separated by centrifugation, and the remaining free AfB1 in the supernatants was quantified by ELISA. The adsorbed amount of AfB1 was determined by difference calculation. Figure 4 and Table 4 show data from two consecutive experiments each carried out in quadruplicate.

Although such high toxin concentrations are not obtained in practice, Figure 4 demonstrates PCT’s high adsorption potential for AfB1, regardless of the respective plant-based beverage. There is no significant difference in the amount of toxin bound per 1 mg of sorbent (far above 150 ng/mg) across all tested matrices (see colored bars of Figure 4). The lower relative proportion of bound AfB1 in the soy drink (Table 4 and gray bar in the middle of Figure 4) was due to the slightly higher initial toxin concentration in these test samples. This could, in turn, be a result of a higher toxin recovery rate in this specific matrix.

Summarizing the results gained from this investigation (Table 4), in soy drink, three-quarters of spiked AfB1 were bound to PCT, while in all other milk substitutes tested, even more was removed by PCT, finally reaching nearly four-fifths in oat drink.

All plant-based beverages showed pH values in the neutral or slightly basic range.

### 2.2. Adsorption of Aflatoxin M1 in Spiked Cow Milk on Purified Clinoptilolite-Tuff

Since AfM1 is a metabolic product of AfB1 generated by the lactating animal, AfM1 can be found in milk from cows and other grazers fed with AfB1-contaminated feed. Hence, the aflatoxin is carried over not only to milk but also to thereof resulting products consumed by humans and animals. So, the first point of interest regarding AfM1 was to determine if PCT can bind the mycotoxin in a complex matrix, as milk is, or if its proteins, natural sugar, and, in particular, fat hinder AfM1 binding to PCT. Preceding tests with PCT and AfB1 in plant-based beverages presented promising results.

The primary experiments were performed in a phosphate buffer at a pH of 6.8 and a test solution at a pH of 1.5. Afterwards, a more complex procedure with artificial juices mimicking human digestion in vitro was chosen to gain results with higher significance for a possible human application.

#### 2.2.1. The Sorption of AfM1 to PCT at Different pH Values

First experiments were performed with either phosphate buffer pH 6.8 or test solution pH 1.5. The concentration of PCT was fixed to 4 mg/mL, corresponding to a daily intake of 2 g in a 500 mL theoretically expected stomach volume. AfM1 spikes were calculated to reach ascending toxin concentrations up to 5000 ng/L, which is a hundredfold of the legally defined limit concentration for AfM1 in milk in the European Union [8]. After mixing the spiked milk with test solution pH 1.5 1:5 (*v*:*v*), AfM1 concentrations up to nearly 1000 ng/L in the test samples could be achieved. The primary objective of the experiments was to saturate PCT with AfM1 and thereby calculate the adsorption capacity of PCT for AfM1. The incubation at 37 °C and 17 rpm took 1 h, simulating the retention time of food in the stomach. The two different pH values of phosphate buffer and test solution, respectively, were necessary to imitate the nearly neutral level of the intestine (pH 6.8) and the highly acidic level of the stomach (pH 1.5); both were blended, corresponding to the European Pharmacopoeia [53]. These conditions should simulate a human digestion process very roughly. Analyses of the supernatants were performed by ELISA [RIDASCREEN^®^ Aflatoxin M1 Art.No. R1121].

**Figure 5:**
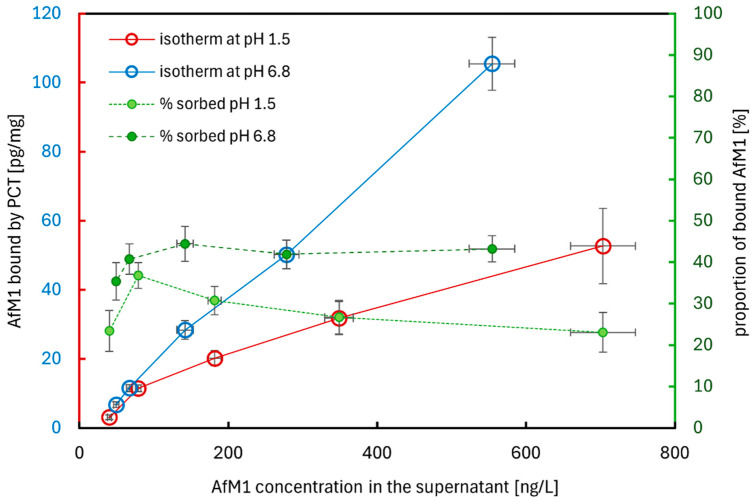
Amount of adsorbed AfM1 in correlation with initial toxin concentration at pH 1.5 (red line) and pH 6.8 (blue line) after 1 h incubation at 37 °C and 17 rpm. The content of AfM1 was determined by ELISA. Each curve represents data from three consecutive experiments carried out in quadruplicate. Standard deviations are depicted for each AfM1 concentration used.

Figure 5 shows the adsorption properties of PCT for AfM1 with increasing mycotoxin concentration in test solution pH 1.5 (red line) and phosphate buffer pH 6.8 (blue line) without further interference factors (like those found in artificial gastric or intestinal fluids). The two different pH values were confirmed by pH electrode measurement.

Although the concentration of AfM1 was unrealistically high, neither at pH 1.5 nor at pH 6.8 was a saturation of PCT achieved. Up to a toxin concentration of almost 100 ng/L in the experimental samples, there was no detectable difference in none of the two reagents corresponding to the amount of AfM1 sorbed to PCT. Higher, but unrealistic, toxin concentrations showed specific patterns of sorption between the individual pH fluids: in the acidic milieu, significantly less AfM1 was bound to PCT than at nearly neutral pH. This discrepancy is in concordance with former experiments applying gluten or peanut protein as absorbent of PCT using the same two fluids with distinct pH values [48,49].

#### 2.2.2. The Kinetics of AfM1 Sorption in Milk by PCT at Different pH Values

**Figure 6:**
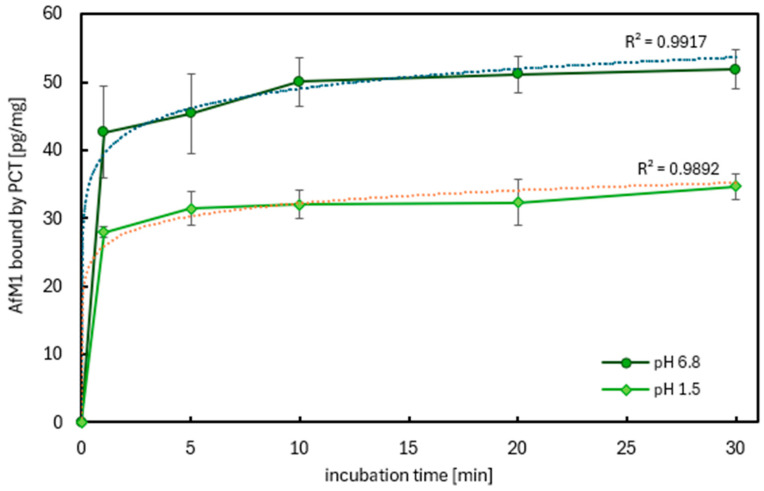
Kinetics of AfM1 sorption by PCT at different pH values (corresponding to stomach pH 1.5 and intestine pH 6.8). The graph represents data of two consecutive experiments for each pH value, both carried out in quadruplicate (continuous lines). Standard deviations are depicted for each time point. The dotted lines refer to the logarithmic trend lines of both graphs, with function for pH 1.5 (red) as y = 2.7411ln(x) + 25.883 and for pH 6.8 (blue) as y = 4.1886ln(x) + 39.397.

Due to the results gained (Figure 5), a more detailed investigation of the kinetics of pH-dependent sorption of mycotoxin AfM1 to PCT was carried out. Once more, the recommended testing solution pH 1.5 and phosphate buffer pH 6.8 of the European Pharmacopoeia [53] were utilized for this purpose. The aim was to analyze the binding velocity of AfM1 to PCT—in particular—whether the reaction proceeds fast enough to be effective within a time frame relevant to digestion. Therefore, the incubation time at 37 °C after addition of PCT (4 mg/mL) to the mycotoxin solution (intended concentration 500 pg/mL, referring to 10 times the legal European limit) was varied, beginning at 1 min to 5, 10, 20, and ending at 30 min, finally. Controls for each time point consisted of AfM1 samples without PCT. Then, all of the specimens were analyzed by ELISA (Figure 6).

As depicted in the diagram, the graphs of two summarized results of each pH value were very close to the coefficient of determination R^2^ = 1, hence logarithmic functions could be used to describe the sorption kinetics.

Sorption took place extremely fast, within the very first minutes. After only one minute, approximately 80% of saturation was reached, which corresponds to about 28 pg AfM1 bound per 1 mg PCT, while a maximum sorption of 35 pg/mg PCT was found after 30 min in the case of pH 1.5. At pH 6.8, after 1 min, about 42 pg AfM1 were sorbed to 1 mg PCT, and a maximum binding of circa 52 pg/mg PCT was detected after 30 min. Once again, there was a discrepancy in sorption capacity between the different pH levels: relating to the daily recommended dose of PCT in humans, 2 g can bind 70 ng AfM1 in a very acidic milieu, as even 100 ng are sorbed in a solution with relatively neutral pH 6.8 to 2 g of PCT.

The higher standard deviations at both pH levels at time point 1 and 5 min might be explained by the fact that the velocity of the sorption reaction at that time was at the highest grade, and so minimal differences between the quadruples lead to relatively big differences. This phenomenon vanished the more the saturation capacity was reached, as can be seen beginning after 10 min of incubation time.

#### 2.2.3. Adsorption of AfM1 by PCT During Simple Artificial Digestion by Stepwise Increase in pH and Desorption of AfM1 from PCT After a 4 Hour Incubation

Creation of a very simple digestion model according to one of the protocols proposed in the European Pharmacopoeia [53] was another aim of the study. As the sorption behavior under constant pH levels (pH 1.5 and 6.8) was defined before, now a stepwise increase in highly acidic pH to an approximately neutral level during a single binding reaction should provide a more realistic access to the performance of PCT as a mycotoxin sorbent. Furthermore, to determine whether the mycotoxin is bound permanently or only transiently to PCT, the AfM1-loaded PCT was subjected to an extended incubation period of 4 h under constant conditions prior to analysis.

**Figure 7:**
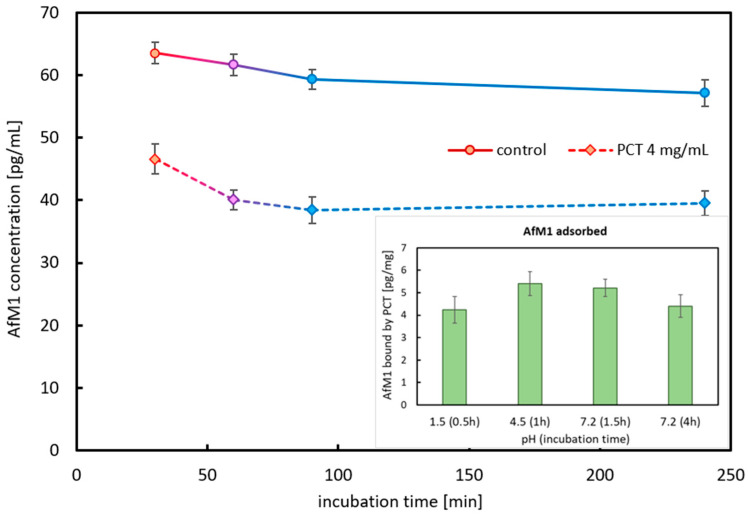
Adsorption/desorption during a 4 h artificial digestion (including a stepwise increase in pH). The lines (gradient colors for changing pH values during the experimental setting) show the unbound amount of AfM1 in samples without (continuous line) and with (dashed line) PCT. The bars reflect the amount of bound AfM1 per mg PCT. Analyses were performed by ELISA. Data and standard deviations of two separate experiments each carried out in quadruplicate are demonstrated.

Starting the adsorption process investigation using cow milk contaminated with 6-times of the legal limit of AfM1 (intended concentration 300 pg/mL) and PCT (4 mg/mL) in the stomach pH simulating reagent (test solution pH 1.5), the pH was elevated in two consecutive steps by addition of TRIS and sodium acetate to pH 4.5 first and then pH 7.2. The process took place at 37 °C (human body temperature) under a gentle rotation (mimicking the movement inside the bowel, 17 rpm) of the samples. Each step lasted 30 min, so that an incubation time of 90 min was finally achieved. The incubation was extended to another 150 min under these conditions to detect any desorption of AfM1 from PCT. Samples without PCT served as controls for each step and time point. Analyses were performed by ELISA (Figure 7). Table 5 shows the rising in the pH value due to the addition of sodium acetate and TRIS. But there was no change in pH caused by PCT, as its concentrations used were too low to be influential.

As depicted in Figure 7, the results were highly reproducible. At the start of the sorption experiment, the concentration of AfM1 was 63.5 pg/mL. Once again, in the highly acidic test solution at pH 1.5, the adsorption capacity was lower than at any other pH level. Approximately 4 pg AfM1 per 1 mg of PCT were bound, contrary to higher pH levels: after 1.5 h of incubation, 5.2 pg/mg of AfM1 were sorbed to PCT. Reflecting the kinetics of the sorption process and the previous demonstrated pH dependency, one can assume that this phenomenon was not due to the longer incubation time but to the changes in the pH milieu.

Interestingly, just a slight desorption could be detected after an extended incubation.

From a purely mathematical point, the consumption of a glass of milk (200 mL), being intoxicated with the legal limit of AfM1 (50 ng/L), could be neutralized by a daily dose of PCT (2 g).

Table 5 shows pH values over 2.0 after 30 min. As the test solution was checked prior to experimental start for pH 1.5, the increase in pH before the addition of sodium acetate and TRIS was due to the mixing of the test solution pH 1.5 with milk (pH 6.75, determined by pH meter).

#### 2.2.4. PCT as an Adsorbent of AfM1 in Contaminated Cow Milk During Simulated Digestion Using Artificial Digestive Juices

**Figure 8:**
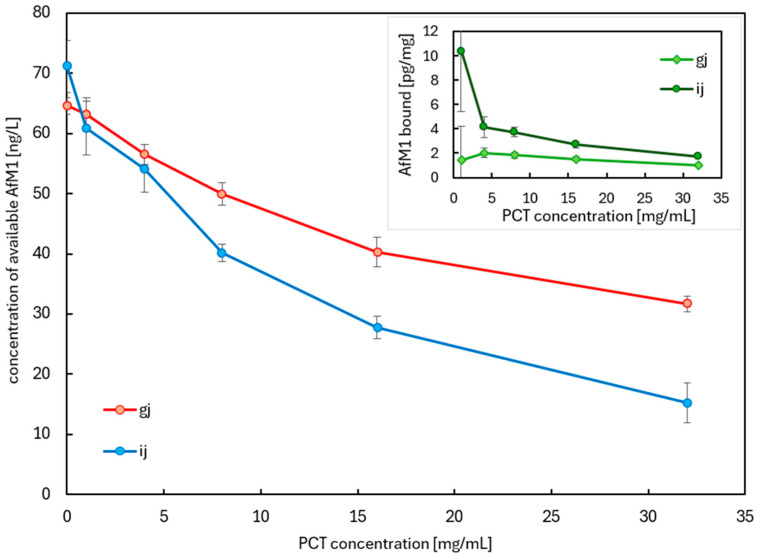
Adsorption in artificial gastric and intestinal juice. Free AfM1 is presented by the red line for gastric juice and the blue line for intestinal juice. The corresponding binding capacity (inserted diagram) is defined by the light green line in the case of gastric juice and the dark green line for intestinal juice. Adsorption is higher in the intestinal juice (pH 6.8) than in the gastric juice (pH 1.2). AfM1 was quantified by ELISA. Data and standard deviations of two consecutive experiments for gastric and intestinal juice, each carried out in quadruplicates per experiment, are demonstrated.

The results described above, gained by simple digestion with test solution pH 1.5 (Figure 7), served as a basis for a more sophisticated sorption study with artificial gastrointestinal juices. Those were prepared as recommended by the European Pharmacopoeia [53]. As a matrix for AfM1, cow milk was used again. Compared to the previous experiments, where cow’s milk was the only complex matrix, the current setup introduced additional components via gastric and intestinal juices, which may potentially interfere with the adsorption of aflatoxin M1 by purified clinoptilolite-tuff. The more complex the milieu, the harder a specific sorption of AfM1 by PCT could be potentially achieved. The following experiments (summarized in Figure 8) were performed to gain insight into the sorption of AfM1 to PCT by occurring of the latter in realistic amounts in gastric or intestinal juice and its performance in detoxifying AfM1.

The intended concentration of AfM1 in the test specimens was fixed to 60 pg/mL (well above the legal limit for milk), while the concentrations of PCT varied from 1, 4, 8, 16, to 32 mg/mL and corresponded to real physiological amounts. Samples without PCT served as controls. The incubation of AfM1 and PCT in cow milk and gastric or intestinal juice took 1 h at 37 °C (mimicking human body temperature), softly rotating (17 rpm).

In the experiments using gastric juice (red line), the AfM1 target concentration of 60 pg/mL was reached almost exactly, and 65 pg/mL could be determined via ELISA. For intestinal juice (blue line), the mean value of AfM1 used corresponded to 70 pg/mL. Both lines show decreasing free AfM1 concentrations corresponding to simultaneously increasing PCT concentrations in the matrices. In gastric juice (light green line), adsorption values of 1 to 2 pg/mg PCT were acquired. In the nearly neutral milieu of intestinal juice (dark green line), those were significantly higher and detected within 2 to 10 pg/mg PCT. Flipped the results to a daily human intake of 2 g PCT, 2 to 4.4 ng AfM1 in the stomach, and 4 to 20 ng in the intestine could be bound, respectively. Regarding the test settings, between 80 mL and 400 mL of cow’s milk contaminated with AfM1 at the European legal limit could be detoxified.

### 2.3. Neutralization of AfM1 by Purified Clinoptilolite-Tuff in Cow’s Dairy Products of Varying Qualities and in Milk from Sheep and Goat

#### Comparative Study of AfM1 Neutralization by PCT in Cow, Sheep, and Goat Milk and Dairy Products

**Figure 9:**
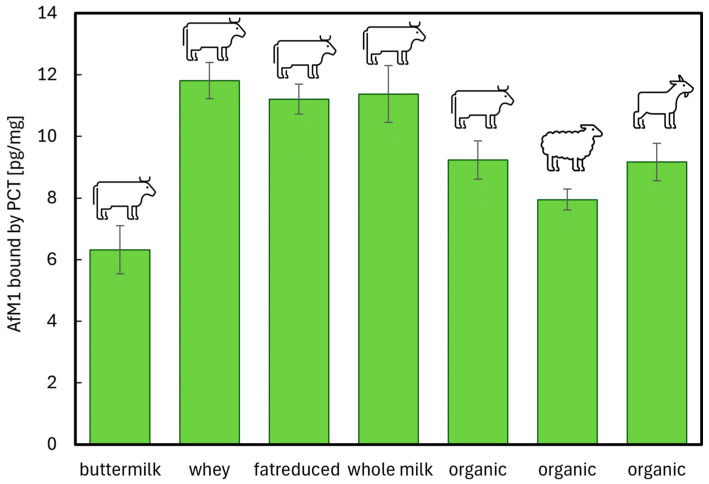
Adsorption of a relevant amount of AfM1 by PCT at pH 6.8 in different types of milk and dairy products at 37 °C with 17 rpm rotation and subsequent measurement via ELISA. The bar chart displays the calculated quantity of AfM1 bound per 1 mg PCT for each respective milk product. The diagram represents data and standard deviations of two consecutive experiments, each carried out in quadruplicate.

Milk is a naturally occurring substance produced by mammals. Hence, its composition varies among species, individuals, and even during seasons. That is why the question was raised, whether PCT, as an aflatoxin-detoxifying agent, shows different adsorption behavior when comparing various cow milk products (like milk, fat-reduced milk, organic milk, buttermilk, and whey) with milk from sheep and goat. Milk and milk products were spiked with 10 times the legal European limit of AfM1 (target concentration 500 pg/mL) and mixed with the testing solution at pH 6.8 to achieve optimal sorption conditions, aside from the matrix. Using a high concentration of AfM1 was necessary for two reasons: the samples (both controls as well as those treated with PCT) had to be diluted after the experiments with dilution buffer 1 + 1, and the values gained had to suit the measurement range (5–80 pg/mL) of the ELISA kit. The samples without (controls) and with PCT (4 mg/mL, actually a daily dose of 2 g suspended in 500 mL gastric fluid) were incubated at 37 °C for 30 min before measuring the content of free AfM1 (Figure 9).

Under the described experimental conditions, the adsorption capacity of PCT for AfM1 in all test matrices (dairy products) was in a comparable range between approximately 6 and 12 pg AfM1/mg PCT. Nevertheless, significant differences between some products were observed. At first glance, the lowest sorption capacity of PCT for AfM1 in the buttermilk matrix stands out. Despite significantly lower fat and protein content compared to, for example, cow’s whole milk, only about half as much toxin was adsorbed in buttermilk. In contrast, no significant differences were detected between cow’s whole milk, reduced-fat cow’s milk, and whey, although these products differ significantly in fat and protein content (Table 6). The adsorption capacities above 11 pg/mg observed here correspond, extrapolated to a daily dose of 2 g PCT, to neutralization (detoxification) of at least 440 mL of milk contaminated with AfM1 at the legal limit.

Compared to conventional cow’s milk, approximately 2 ng/mg less toxin was adsorbed in organic cow’s milk, further indicating that total fat and protein content per se are not the decisive factors for the adsorption of AfM1 to PCT. The adsorption capacity in organic goat’s milk was at the same level as organic cow’s milk and higher than that in nutrient-rich organic sheep’s milk.

In summary, purified clinoptilolite-tuff binds significant amounts of aflatoxin M1, even within complex matrices such as dairy products. Total fat and protein contents are not direct indicators of binding capacity. Rather, the qualities of the proteins or other components altogether could have an influence on the sorption process. For example, buttermilk has significantly higher viscosity due to the coagulated proteins (mainly casein) resulting from fermentation processes. This could be the reason for the observed reduced sorption capacities compared to micro-filtrated, relatively thin whole milk.

## 3. Discussion

### 3.1. The Complexity of Mycotoxin Science

Mycotoxins are defined as chemical hazards of fungal origin. From the end of the exponential growth phase to the beginning of the stationary phase, molds start to generate their toxic secondary metabolites. This can happen on the field (crop) but also during storage of the raw material (corn, grain), respectively, in the intermediate product (flour) or in the final food product itself [54].

The susceptibility of mammals to mycotoxins depends on many factors regarding the single individuum—it is a combination of bioavailability, type of toxin (single or co-occurrence), amount and concentration ingested daily, the continuity of the consumption, weight and physiological state, health, age, sex, species, nutrition, milking time, etc. [12,14,54].

Masking of aflatoxins (also known as hidden aflatoxins) is a known phenomenon, in which they become undetectable within the matrix due to interactions that hinder their analytical detection. This can either happen due to binding of aflatoxins to other components like emulgents, fats, sugars, salts, vitamins, minerals, and synthetic additives in the complex composition of the matrix—some of the used analytical techniques are not able to destroy these connections and the aflatoxins remain undetected—or it is provoked by the action of the digestive tract like the gastric acid or enzymatic processes, so that the toxin is partially degraded and unrecognizable by the method applied. In the case of bioaccessibility, those toxins are not considered as potentially susceptible to being adsorbed by the intestine [1,6]. It cannot be definitely excluded that the resulting mycotoxin fragments do not affect the organism. Hence, binding of the remaining toxin pieces by PCT could be of advantage for the organism. Results gained in experiments performed for this publication indicated that with rising PCT concentration, the bioaccessibility of AfB1 was reduced due to PCT binding.

### 3.2. Aflatoxins as a Global Concern

Aflatoxins are among the most prominent representatives of mycotoxins. Until today, they are feared impurities as their actions are manifold on the health of the human and animal body. Acute and chronic exposure to different toxin concentrations often leads to dysfunctions of many organs, but might also influence the embryonic development and growth of children and young animals [5]. In 23% of the investigated baby food products worldwide, aflatoxin contamination was verified [15,55]. Aflatoxins also provoke various cancer types, among which the hepatocellular carcinoma is one of the major types of liver cancer. Developing countries contribute over 80% of all cases, and 5–28% of all hepatocellular carcinomas may be attributed to aflatoxin exposure. Hence, dietary exposure to aflatoxins is considered the second largest environmental risk factor for liver cancer development after infections with viral hepatitis B and C. Unfortunately, suffering from hepatitis B or C acts synergistically with aflatoxins [4]. Consequently, an effective, low-cost prevention of aflatoxin impact with uncomplicated use is needed.

Aflatoxin-producing fungi need a minimum temperature of 6–8 °C for their growth; their optimum growth condition is between 36 °C and 38 °C, but they can even proliferate at maximum temperatures of 44–46 °C. The toxin production starts at 12 °C, reaches its maximum at 30 °C, and proceeds to 42 °C at maximum [5]. The experiments presented here were performed at 37 °C, which is not only referred to human body temperature, but also desirable for *Aspergillus* species to produce aflatoxins. A pH of 5 combined with a relative humidity of 80% is the best condition for the production of approximately 20 different isocoumarin derivatives, as aflatoxins are chemically defined. Because aflatoxins can persist in heat and pH ranges from 3 to 10 [54], those toxins largely withstand food processing. For instance, the amount and toxicity of aflatoxin M1 are negligible affected by pasteurization at 72–75 °C or ultra-high temperature processing at 120–150 °C [14]. Experiments showed that PCT has an impact on the concentration of free available AfB1 and AfM1 at human body temperatures and at both pH values (1.5 and 6.8) tested (Figure 2, Figure 3, Figure 5, Figure 6, Figure 7 and Figure 8). Only small differences concerning the recovery rate of aflatoxins were detected at the two pH values up to 4 h (Figure 7).

### 3.3. Aflatoxin B1 in Plant-Based Beverages

AfB1 is assumed to be the most toxic aflatoxin. When plant-based beverages are consumed as an alternative to cow’s milk, the overall intake of AfB1 via food is elevated because in plant-based beverages, AfB1 is not transformed to the potentially lower toxic AfM1, as this happens in animal milk. Regarding chronic exposure, this might be of special importance by increasing the likelihood of health impairments [20].

Plant-based beverages present complex matrices due to their high contents of proteins, carbohydrates, lipids, and fibers. Their raw materials are prone to fungal infection and, hence, to mycotoxin contamination. The annual global growth rate of plant-based beverages is near 9% [19]. Pavlenko et al. indicated that many studies were dedicated to mycotoxins in teas as well as juices composed of fruits and vegetables, while plant-based beverages consumed as an alternative to animal milk substitution were less regarded [10]. For this paper, experiments with complex matrices like almond, hazelnut, soy, oat, and cashew drinks were not only performed in test solution pH 1.5 and phosphate buffer pH 6.8 (Figure 2 and Figure 4), but also in artificial gastric and intestinal juices. Although various ingredients are present in both drinks and artificial juices, PCT could efficiently remove, particularly the added aflatoxin B1 (Figure 3). Within the zeolite family, clinoptilolite shows a relatively high Si/Al ratio. This favors the binding of aflatoxins in aqueous solutions, as there is less competitive hygroscopic effect towards the binding to clinoptilolite [34]. Furthermore, the literature indicates that acidic treatment of zeolites with sulfuric, hydrochloric, nitric, or acetic acids leads to partial dealumination of the lattice, thereby increasing the available surface area. Elevating the Si/Al ratio additionally promotes the adsorption efficiency [34]. The performed experiments indicate that, for AfB1, sorption was substantially higher at pH 1.5, resulting in an approximately threefold increase in the calculated maximum adsorption capacity under acidic conditions compared to near-neutral pH, as found in phosphate buffer (pH 6.8). This effect was incisive in milk and dairy products (Figure 5, Figure 6, Figure 7 and Figure 8), which might be due to the oil-in-water emulsion milk is based on, as well as on the composition of its matrix. Due to the acidic pH of 1.5 in the test solution and gastric juice, milk coagulates, leading to relatively big chunks that encompass the small PCT particles, thereby preventing a vivid interaction of the oil-in-water emulsion with the PCT particles normally found in milk without flocculation at a pH of 6.8. Clinoptilolite displays preferences for certain cations like calcium, kalium, sodium, and magnesium. Those are found located in the different channels of the framework [34]. As they are also found in milk, their trapping might weaken the acidic effect. This is in accordance with the findings of Moore and colleagues, who tested plant-based beverages compared to animal milks of cow and goat on their content of total proteins, lipids, amino acids, and minerals. They described that almond-, oat-, rice-, and coconut-based beverages were of lower nutrient quality than both types of milk. Moreover, antinutrients—which are naturally occurring in plants—can even lower the already weak nutrient quality of plant-based beverages as they reduce the bioavailability of those nutrients. The only exception was a soya-based beverage that was rather similar to animal milks regarding the protein content and amino acid composition and showed also a high mineral content [18].

Although the European Commission regulates the total level of aflatoxins B1, B2, G1, and G2, and there is a maximum level of AfB1, the bioavailability does not always correspond to the fixed levels. When testing the bioaccessibility of AfB1, AfB2, AfG1, and AfG2 in commercially available plant-based beverages composed of either almond, oat, rice, and soya, Romero-Sánchez and colleagues found different matrix effects in association with the type of toxin. The highest level was obtained for AfB2 (82–92%), followed by AfG2 (32–76%), and AfB1 (28–50%). The lowest value corresponded to AfG1 (15–30%). Interestingly, for AfB2 and AfG1, the type of plant origin is rather irrelevant; contrary, AfB1 and AfG2 bioavailability depends strongly on the composition of the matrix. This is of particular importance as all four aflatoxins show high structural similarity. AfB1 has its major bioaccessible value in almond drinks, which contain a lot of fats, whereas the lowest value was gained in beverages with a high carbohydrate proportion (oat, rice). Soya drinks showed an intermediate value corresponding to similar fat content to almond drink and high protein content like oat and rice drinks. Opposite results were gained for AfG2, which has higher bioaccessibility in oat and rice drinks, which is contrary to almond drinks. Soya drinks gave intermediate results. Impressively, the results show the importance of fat, carbohydrate, and protein content for the impact of aflatoxins on the human and animal body. AfB1 might be considered the most lipophilic aflatoxin tested in the experiments [6]. Covering a broad spectrum of various plant-based beverages and considering their main components due to their origin from different plant families (Table 1), for this paper, the following drinks were used: As representatives of high-fat-containing plant drinks, hazelnut, almond, and cashew were used. For containing high amounts of carbohydrates, oat was chosen, but rice would also have been fine. Finally, as a high-protein drink, soy was present. AfB1 bound to PCT in all plant matrices used for the experiments. Despite a minor matrix effect, PCT exhibited high efficacy in neutralizing AfB1. Figure 4 and Table 4 show approximately the same level of AfB1 binding in all drinks used, which corresponds to a calculated adsorption capacity of PCT of approximately >150 ng/mg (the AfB1 concentration implemented at the experimental start was 200 pg/mL, while it was reduced after incubation with PCT to about 50 pg/mL at the ending of the test). The experiments of Romero-Sánchez and colleagues were performed in digestive fluids (salivary, gastric, duodenal), which were quite similar to the artificial juices used in this publication. Also, pH values as well as agitation (35 rpm) and incubation temperature (37 °C) corresponded well. Only the incubation time (2 h) lasted clearly longer [6]; however, this is likely to be unproblematic for comparison of the results gained, as the neutralization of aflatoxins B1 and M1 occurred rapidly, with the majority of the available toxins being bound to purified clinoptilolite-tuff within the first few minutes (Figure 6 and Figure 7).

Butovskaya and colleagues examined 42 plant-based beverages (soy, oat, rice, and almond) for—amongst other things—contamination with toxic trace-elements (lead, cadmium, arsenic, nickel, chromium) and mycotoxins (AfB1, AfB2, AfG1, AfG2, DON, ZEA, FB1, FB2, T2, HT-2, OTA). Data on toxic trace-elements suggest that plant-based beverages contribute low percentages to the dietary exposure to lead, cadmium, and chromium (III), and there are concerns about inorganic arsenic detected in rice drinks and nickel derived from soy and oat drinks [56]. The plant-based beverages used in this work consisted of 2% almond, 2.5% hazelnut, 5.5% oat, 6.5% cashew, and 7.1% soy. Thus, due to the low concentration of raw plant material, the toxin concentration of AfB1 was estimated to be in a similar order corresponding to the concentration of AfM1 in milk. The initial toxin concentration of AfB1 for the binding experiments with different kinds of plant-based beverages was set to 1000 ng/mL, resulting in 200 ng/mL in the samples (Figure 4), while that of AfM1 in milk and milk products was 300 pg/mL, resulting in 60 pg/mL in the samples (Figure 9). These concentrations should push PCT to its limits, which was necessary for the calculation of the maximum binding capacities. Moreover, PCT already demonstrated a heavy metal binding affinity before, so that both toxins and venomous metals might be captured at the same time by PCT [47].

### 3.4. Aflatoxin M1 in Dairy Products of Different Origins

Among all dairy products, milk is the most widely traded and processed, with per capita consumption reaching up to 150 kilograms per year. AfB1 is transformed with a conversion rate of 0.3–6.2% in the digestive system of the lactating animal to AfM1 [54]. An intake of <40 µg AfB1 daily per dairy animal forces an excretion of approximately 0.05 µg/kg AfM1 in milk. Permitted levels of AfM1 were established for milk and dairy products in various countries. Even at low concentrations (≤1 µg/kg/day), AfM1 shows toxic effects. Contrary to AfB1, AfM1 does not require cytochrome P450 activation to be cytotoxic; hence, cells are directly affected by the action of AfM1 when achieving contact [54,57]. Interestingly, AfM1 was highly absorbed in differentiated Caco-2 cells, which portray a model of human intestinal enterocytes [57]. Moreover, microorganisms in the cattle can turn AfB1 into the highly venomous aflatoxicol [54]. PCT might bind aflatoxins in the gastrointestinal tract of livestock and save them from being ingested via the enterocytic pathway.

Generally, aflatoxins in milk are mainly present in the milk serum (~46.5%) and in casein (~48.5%), while only a small proportion is found in the fat fraction (~5%) [11]. AfM1 primarily binds to casein, which is, with 80% the largest proportion of total protein content in cow milk [23,58]. In some milk derivatives (e.g., yogurt and cheese), AfM1 is enriched, due to association with the protein fraction, with 3–5-fold compared to milk [57]. As AfM1 is aberrant in amount in the different milk fractions, not only various kinds of milk products (spiked with AfM1) were tested for this work, but also the content of AfM1 was defined at changing pH levels (1.5, 4.5, 7.2, Figure 7—small, inserted diagram and Table 5). Obviously, PCT bound AfM1 at all levels tested and in all matrices used (Figure 8 and Figure 9). However, in the extremely viscous buttermilk (low fat content, Table 6), reduced affinity between PCT and AfM1 was revealed (Figure 9).

In 2015, about 70% of 960 raw cow milk samples from Punjab, Pakistan, were contaminated with AfM1 at levels exceeding the permission levels of the United States of America (500 ng/L). The contamination with AfM1 of milk was also reported—amongst others—from Brazil, Croatia, Korea, Morocco, North Africa, Portugal, Serbia, Slovenia, Spain, Sudan, Syria, and Turkey [24]. In Yazd province, Iran, raw milk from cows, camels, sheep, and goat was analyzed for AfM1 contamination. In all milk types, the toxin was found, but camel milk displayed the lowest detection rate, and none of its samples exceeded the legal limit of 50 ng/kg. The concrete levels were as follows (detection rate/over the legal limit): cow 46.5%/15.4%, sheep 21.6%/11.5%, goat 20.1%/9.15%, camel 4.03%/0%. When comparing the levels of AfM1 in cow milk from Iran to those described in Croatia, France, and Portugal, they are higher than in countries of the European Union, where regular monitoring programs are installed. Regarding sheep and goat milk, the results of Iran were comparable to other countries like Turkey, Syria, Egypt, and Pakistan. Once again, values from European countries—Italy, Croatia, and Greece—were considerably lower. Similarly to cow milk, the milk of sheep and goats was most contaminated in winter. Contrary, camel milk was contaminated with low AfM1, which might be due to their eating habits as camels graze throughout the year and seldom receive stored grains and concentrated foodstuffs like other animals in Iran [59]. In Europe, 50 ng/kg AfM1 in milk is permitted, whereas in some other countries, like the USA, the limit is set 10 times higher. To mimic a more realistic toxin concentration in countries massively affected by mycotoxin contamination, one goal of this work was to use a concentration 10 times higher than those allowed in Europe (Figure 9). Moreover, this experimental setting should also reveal the maximal binding capacity of PCT towards AfM1. A single daily dose of PCT (2 g) could neutralize 10–100 ng AfM1 (Figure 6 and Figure 7). Experiments were performed with sheep and goat milk, as well as with various cow milk products (Figure 9). Adsorption was assessed by incubating a constant PCT concentration with increasing AfM1 toxin concentrations at the different pH values of 1.5 and 6.8 (Figure 5). As shown in these experiments, as well as in former analyses [48,49], offering very high amounts of toxin or allergen at low pH matrices has an adverse influence on the binding compared to nearly neutral pH ones. However, PCT bound the milk toxin at the pH levels without any deviation (Figure 5), resulting in 100 ng/L AfM1 toxin in the supernatant.

A study performed in Malaysia revealed the contamination with aflatoxin M1 of 199 out of 444 humans who consumed cereal products. In the 37 urine samples, the AfM1 value exceeded the limit for detection of 0.64 ng/mL, ranging from 0.64 to 5.34 ng/mL [60]. When taking into account that 0.3–6.2% of AfB1 are converted to AfM1, one can calculate that 5.34 ng/mL AfM1 can be derived from a range of 86–1780 ng/mL AfB1. Depending on the pH of the medium (Figure 2), less than 1 mg of PCT was adsorbed between 5000 and 14,000 ng/mg of AfB1 in gastric and intestinal juice, respectively. This is in accordance with the work of de Lima Schlösser and colleagues, where they described the sorption of AfB1 to clinoptilolite at two different pH values (pH 3 and pH 6) in an in vitro model. The Langmuir model resulted in an adsorption capacity of 8.6 mg/g at pH 3 and 2.3 mg/g at pH 6 [61]. This is in concordance with the results obtained in this work: 14 mg/g at pH 1.5 and 5.0 mg/g at pH 6.8 (Figure 2). Hence, theoretically, the highest intoxication concentration (1780 ng/mL) of AfB1 described by Sulaiman and colleagues could be easily sorbed to PCT.

### 3.5. Strategies Against Aflatoxin Exposure

#### 3.5.1. Prevention and Avoidance

Good Agricultural Practice is an admirable way to keep contamination low. But the best actions taken cannot guarantee that no molds are growing, and no toxins are synthesized [12].

Regarding aflatoxin levels, the European Union established the ALARA (“as low as reasonably achievable”) principle. The EU maximum limit for AfB1 in dairy feed is regulated at 5 µg/kg (EU directive 2002/32) [62] and is set for milk at 50 ng/kg [7]. In the United States of America, Brazil, and China, the limit is set to 500 ng/L milk. In 2002, about 100 countries restricted the AfM1 content in milk and milk-based products [24]. Contrary to industrial countries, developing nations (mostly localized in the hot zone between a latitude of 40° N and 40° S of the equator) depend on this food or cannot manage to test products on mycotoxins adequately, leading to severe health problems of their citizens, even more intensive when malnourishment is prevalent [3]. Especially in such countries, a sufficient, practical, and easy to use, cost-effective, and sustainable decontamination method/technique/solution is needed, as avoiding the consumption of toxic food and feed is impossible [2].

#### 3.5.2. Detoxification/Decontamination

The intrinsic complexity of recognizing and separating contaminated from clean crops forces the detoxification process towards decontamination of (liquid) foods rather than purifying the original raw materials [11].

Miscellaneous detoxification methods of animal feed as well as of human food were developed, and improvement is of great interest. The techniques include physical, chemical, and biological processes, which are—for feedstuff—defined in the EU Regulation 2015/786 [63]. However, in the Commission Regulation (EU) 2023/915 of 25 April 2023 [8] on maximum levels for certain contaminants in food and repealing Regulation (EC) No 1881/2006 [9], for human foodstuffs, no chemical decontamination treatment is allowed.

##### Chemical Mitigation of Aflatoxin Contamination

A way of reducing, destroying, or inactivating aflatoxin M1 in milk is by chemical approaches. However, it is nearly impossible to detoxify aflatoxins without reducing the nutritive value and palatability of milk. Also, a lot of parameters must be regarded—like reaction time, temperature, and moist—which is time-consuming and expensive, especially as there are several necessary cleaning steps involved in the procedure to get rid of toxic byproducts of the single processes [22].

##### Physical Methods to Reduce the Aflatoxin Content

- Temperature

The AfM1 contamination in milk was reduced by boiling at 100 °C for 10 min, thereby decreasing the AfM1 levels by 12.55%, whereas microwave radiation at high energy levels for 2 min lowered them by 42.96%, which is in concordance with results gained by other researchers for these processes [25].

- Sorbents

Adsorption of mycotoxins by nanoparticles, nanocomposites, magnetic-activated carbon, as well as various kinds of adsorbents, is extensively tested currently. Sorbent agents with the ability to bind multiple toxins are of particular interest due to their potential to act within the mammalian gastrointestinal tract, thereby preventing the systemic distribution of these toxins throughout the organism. To this group belong activated carbon (charcoal) and some established minerals that have been implemented in animal husbandry over decades, like zeolite, bentonite, montmorillonite, vermiculite, nontronite, hydrated sodium calcium aluminosilicate (HSCAS) derived from bentonite, sepiolite, and diatomite [5].

The reduction in nutrients by mycotoxin sorbing substances is not desired. A study by Di Natale, Gallo, and Negro targeted several carbonaceous sorbents, zeolites, as well as bentonite and clinoptilolite with regard to their specificity for aflatoxins and their unwanted unspecific binding of appreciated ingredients. Generally, all tested materials revealed more or less that the adsorption treatment reduces the content of organic acids, chlorides, lactose, and total protein, and increases the pH of ultra-heat-treated whole cow milk. Activated carbons exhibit the highest removal efficiencies for aflatoxins due to their large surface area, appropriately sized pores for toxin adsorption, and the strong affinity between aflatoxin molecules and the aromatic structure of the carbon material. However, the filtration of important nutrient substances is also high [11]. A comparative test with the use of a natural Greek clinoptilolite and activated carbon (5 g/L, each) toward the adsorption of methylene blue (250 ppm) revealed a much faster sorption of clinoptilolite than charcoal. The authors referred to a chemisorption process with regard to the action of clinoptilolite, which was also suggested by experiments of another research group. Compared to activated charcoal, clinoptilolite offers a sustainable and cost-effective alternative [34].

Dommergues and Mangenot published already in 1970 that clays can adsorb large molecules like antibiotics, pesticides, and products of microbial metabolism [64]. Then, in 1978, Masimango and colleagues released an article about the decontamination of AfB1-contaminated liquid media by various phyllosilicates. Desorption experiments revealed that only chloroform extraction led to the release of aflatoxins from some clays, whereas others released only a very minor amount. It became clear that aflatoxins could not diffuse again in solution but bound irreversibly [37]. Desorption experiments performed for this paper gave the same results (Figure 7). Testing different fractions of phyllosilicates showed the higher sorption capacity of fine powder compared to coarse-grain size [37]. Previous experiments with PCT and gluten showed a similar outcome [48]. Described as being safe for human application and as chemically inert materials, clays should not noticeably interfere with food quality [37]. From ancient times onward, bentonite has been used as a dietary supplement. Clay materials—especially bentonite—have been employed in the food-processing industry for decades now [5,32]. Bentonites are chosen for their special physical and chemical characteristics, including surface specificity and enlargement (when achieving contact with water, bentonite can swell up six times its original size), adsorption, cation exchange, low cost, high safety, and rheological and colloidal features. Haman and colleagues proposed that the sorption of AfM1 is primarily based on the properties of the bentonite; hence, the composition of the sorbent could be a marker for its detoxifying ability. AfM1 is bound stable to bentonite [65], which is in correspondence to the results presented in this paper (Figure 7). Various clays for the adsorption of aflatoxins are already commercially available, and the European Union has approved bentonite as a feed additive for the reduction in AfB1 contamination. Additionally to mixing feed with sorbents, another approach is to decontaminate milk directly by the addition of (clay-based) sorbing minerals [7]. Efficient mitigation under the EU standard limits of 50 ng/kg of AfM1 in milk by bentonite was reported. Alteration of milk’s nutritional content was moderate, and the bentonite residue of 0.4% in milk was no concern for human health [7]. Clays, when used for detoxification, sediment on the bottom and build a pellet after some time. So, some residual minerals will stay in milk. In the USA, the content of bentonite in food is regulated by the FDA without a definition of the final concentration. In the EU, 5% for use in foodstuffs is tolerated [31]. PCT, as a dietary supplement or dietary ingredient, could be used either for direct binding of aflatoxins in food or after food intake. Its application is versatile, and its positive effects were demonstrated in several human studies [66,67].

As early as 1982, Applebaum and Marth described in vivo experiments—inspired by Masimango and colleagues and the fact that AfB1 and AfM1 possess similar structures—with fistulated Holstein cows fed twice daily with AfB1-contaminated feed. Raw whole milk, naturally contaminated with AfM1, was mixed with bentonite in several concentrations, indicating that most of the AfM1 was sorbed to bentonite at room temperature. The sorption behavior was described as a continuous increase with rising bentonite concentration. Further analyses of the milk showed that at the highest bentonite concentration (20 g/kg), only 5% of the total protein amount was lost when compared to controls [38]. The experiments with PCT also showed a constant increase in bound aflatoxins B1 and M1 over time, even in relatively high complex matrices (Figure 6 and Figure 7). Furthermore, clinoptilolites from different pits exhibit various mineral compositions and thus also some versatility in their sorption characteristics. As purified clinoptilolite-tuff (PCT) originates from a methodologically validated process, its quality and composition are constant [46]. The addition of either 0.5% or 2% of bentonite to naturally AfM1 contaminated milk (exceeding 0.05 µg/kg) decreased the toxin level by 90% at both concentrations [32]. Bentonite has—like clinoptilolite—a negative charge, which enhances its adsorptive properties by ion exchange. This is why bentonite has the ability to reduce AfM1 directly in naturally contaminated milk, but can also be used as a feed additive, then reducing the AfM1 levels indirectly by binding AfB1 in the gastrointestinal tract of the lactating animal. When AfB1 is attached to bentonite in the gastrointestinal tract, it is excreted naturally. Furthermore, bentonite is described as having a low impact on the nutritional properties of milk. Besides cows, bentonite was also used for reduction in “milk toxin” in camels, goats, and sheep [4,22,65]. In the experiments performed for this paper, various types of milk derived from miscellaneous animals like sheep, goat, and cows were used. Even though no animal experiments were performed and merely the contaminated milk of these species was utilized, a significant reduction in AfM1 was detected (Figure 9).

In a poultry model among three different smectites (montmorillonite, beidellite, beidellite-Ca-montmorillonite—all of them being phyllosilicates), one zeolite (a tectosilicate consisting of 80% clinoptilolite, which is consistent with PCT) was examined for its detoxification capacity of AfB1. The surface structures and morphology were determined via scanning electron microscopy (SEM) and revealed that the zeolite used consisted of aggregates (10–60 µm) and of spherical particles (1–5 mm), while its cation exchange capacity was found to be 38.29 cmol(+)/kg. For comparison, PCT consists mostly of particles with a diameter of around 3.1 µm and a cation exchange capacity of >75 cmol(+)/kg. Ultra-performance liquid chromatography with 4 h of retention time was used to quantify AfB1 in this in vitro poultry gastrointestinal model. The zeolite sorbed about 81% of AfB1, which refers to 0.161 ± 0.0004 ng/kg, in the small intestine compartment. Additionally, the trace inorganic nutrient adsorption was defined—for the zeolite, it was Fe 58.5 ± 0.1%, Se 58.0 ± 0.7%, Zn 38.0 ± 0.3%, Mn 35.0 ± 0.8% in the in vitro gastrointestinal model for poultry [30]. The adsorption capacity of the zeolite and smectites depended on various factors like the number of active sites located on the interlayer, basal planes, surface, pores, edges of the particles, and exchangeable cations. The adsorption of AfB1 by clinoptilolite was influenced by functional groups and inorganic cations from the external surface, while its higher sorption capacity compared to smectites was explained by the presence of a fairly ordered system of macro-, meso-, and micro-channels. The loss of trace nutrients can be detrimental for the organism; thus, sorbents should have a lesser or non-existent effect on the concentration when used as a food or feed additive. In this study, the zeolite’s affinity towards the elements tested was in the middle range compared to the smectites [30]. Interestingly, the adsorption capacity between smectites and clinoptilolite varies as results of different research teams show. This might be due to the variable composition of the single minerals as well as to the non-identical media, pH values, concentrations of Ca^2+^ and K^+^ ions, etc. [30]. Smectites were also tested in human nutrition studies for the elimination of aflatoxins. However, the crystal–chemical variability of this element complicated the prediction of adsorption capacity [31].

Experiments with a modified clinoptilolite testified to the binding and sorption inhibition of AfB1 and ochratoxin A in the gastrointestinal tract of broilers. Their health status and performance parameters revealed both the improvement of productive performance as well as the decreased levels of mycotoxins in the broilers’ tissue [68]. Another research team analyzed the efficacy of 4 g/kg of either bentonite or zeolite in reducing 0.25 mg/kg AfB1 toxicity in broilers. The production performance, as well as intestinal and hepatic health, were used as indicators. Protective effects towards AfB1 were observed [69].

HSCAS is used as a protector for the gastrointestinal tract as it possesses a great specific area and sorption ability—this is in concordance with the action described for PCT. When included in the diet, HSCAS could save animals from different species (like rodents, chicks, turkey poults, ducklings, lambs, pigs, mink, and trout) from the toxic effects of AfB1, and it also reduced the amount of AfM1 from dairy cattle and goats, as confirmed by several trials [70]. HSCAS adheres to the mucosa of the gastrointestinal tract and helps to avert damage [2].

The mitigation of aflatoxicosis in animals is currently achieved by the addition of several alumosilicates to their feed. Besides mycotoxins, they are used to bind other toxins, phytotoxins, enterotoxins, bacteria, and viruses, which hinders their uptake through the gastrointestinal tract [30]. For most organs, aflatoxins have detrimental effects, especially on the liver. This organ is also the main target for heavy metals like lead, cadmium, nickel, arsenic, chromium, and mercury [4]. In vitro as well as in vivo experiments already gave first indications that aflatoxins and heavy metals show additive consequences [4]. PCT has already been proven to sorb lead, cadmium, nickel, cesium, barium, and thallium in vitro [47,71,72] and lead also in human studies [66,67]. Hence, binding of all or at least one toxic component could make a massive improvement for the individual affected.

Decontamination of low-, medium-, and high-level radioactivity wastes is performed with natural aluminosilicates, such as clays and zeolites. Clinoptilolite and clinoptilolite-tuff proved the ability to decontaminate various radioactive ions like americium, cobalt, strontium, cesium, samarium, europium, and uranium [73]. Sterba and colleagues used in their experiments products with high clinoptilolite content (~90%), in contrast to another product with less content (~60%)—in comparison, PCT contains at least 80% or more clinoptilolite. When assessing the adsorption rate and its dependence on physical factors with regard to two of the most hazardous radionuclides for humans, cesium and strontium, it became obvious that the content of clinoptilolite in the sorbent was of decisive influence, as the more clinoptilolite in the product was available, the more adsorption of cesium and strontium was gained. Furthermore, a smaller particle size supported more surface area necessary for the binding of isotopes [71]. While performing sorption experiments with PCT in the case of gluten neutralization, the fine fraction of PCT clearly showed a higher adsorption rate than the coarse one [48]. A wound dressing composed of PCT-doped calcium–alginate mixed with cellulose fibers was tested for its decontaminating action in radiolytically contaminated environments. Again, cesium and strontium were used as radiotracer material in both aqueous and artificial wound fluid exudate, as they are the principal radiolytic contaminants from fission-related radionuclide releases. Experiments were performed at 40 °C to mimic human body temperature. A divergence of the molar capacity 4:1 of PCT for cesium and strontium was described [72], which is quite similar to the maximum adsorption capacity of a Bulgarian clinoptilolite for cesium and strontium as 5.7:1 [74]. The PCT containing wound dressing adsorbed cesium and strontium in both liquids with only a slight difference, and it was shown that most of the cesium sorption occurred within 30 min [72]. Before, it was already described in the case of high proportion clinoptilolite containing products that the sorption of cesium reached saturation after 10 min, while that of strontium took more than 48 h [71]. For PCT, a fast sorption reaction was observed within only a few minutes for aflatoxins (Figure 6 and Figure 7) but as well as for *Clostridioides difficile* toxins [50], gluten [48], and peanut allergens [49]. PCT also showed binding of heavy metals [47,66]—a characteristic typical for zeolites [73]. A former clinical study using PCT in wound treatment proved its excellent compatibility [75].

##### Biological Methods

Biological methods to mitigate mycotoxin contamination are very product-specific and need time to act, while physical and chemical ones are more versatile in application and are generally faster in their effectiveness [5].

The use of microbes like bacterial strains and yeasts (either individually or in combination) as microbial decontaminators of AfM1 is critical, as when used alive, they need incubation time and a specific temperature and pH range. Thus, often heat-killed cells are applied, which advantageously do not influence the product by their metabolism. AfM1 attaches to cell wall components, the peptidoglycans and polysaccharides. However, there are some reports that the binding is reversible and AfM1 is set free (to a high percentage). Moreover, the binding of lactobacilli and AfM1 varied not only between species but also within different strains, which indicates a high specificity and less versatility of the method. Also, the different legislation between countries on the total amount of viable cells in milk and milk products may concern the consumers of dairy products. Furthermore, bacteria and/or yeasts can proliferate in the product uncontrolled and, hence, spoil the milk. Therefore, a mechanism for microbial removal is needed, which can be expensive and cause loss of important milk components that might be re-supplemented afterwards. Another aspect of this method is that the concentration of AfM1 in milk is not constant, and so the content of microorganisms needed is flexible [12].

In addition, the biotransformation of biodetoxification is studied. Here, microorganisms (bacteria and fungi) and/or their purified enzymes can act on aflatoxins by catabolizing the molecules, by transformation, or by cleavage to mitigate the toxicity of or produce non-toxic final products. But reports point out that this is performed with varied efficiencies, and their utilization in the food industry is rare due to long incubation times, spanning 3 days; incomplete degradation; non-adaptation to typical food fermentations; and culture pigmentation, and some strains used may even produce AfB1 under varying conditions [7].

A different approach when using microorganisms is to use probiotics. Most interesting for this are lactic bacteria as *Lactobacillus*, but also *Streptococcus*, *Pseudomonas*, Bifidobacteria, and *Burkholderia*, and yeast strains of *Saccharomyces*, as well as nontoxigenic *Aspergillus* ssp. The detriment of probiotics is their sensitivity to pH and temperature; minerals utilized for detoxification are independent and stable in their action against these factors [22]. In a mouse model of DSS-induced acute and chronic colitis, the use of PCT not only helped to reduce the symptoms of the disease, but the analysis of the animals’ microbiome revealed that the treatment with PCT left the *Firmicutes* unaffected, and the *Bacteroidetes* were restored—the latter being an indication of the beneficial action of the PCT [70].

Enzymatic degradation is another way to reduce aflatoxins with relatively minor impact on food and feed. As currently post-harvest methods to mitigate aflatoxin content include only physical techniques (sorting and sorption), it offers an addition to the methodical pool. For example, all detected degradation products of AfB1 exhibited a higher polarity and excretion rate via urine and feces, but lower toxicity (like AfQ1, epi-AfQ1, AfB1-diol, AfB1dialdehyde, AfB2a, AfM1) when treated with Ery4 laccase (a copper-containing enzyme). However, they retained the ability to generate adducts showing residual toxicity. Moreover, there is a risk in food and feed that single components compete for, or adsorb the enzyme, leading to a lowered efficacy, which can further be affected by a higher viscosity of a real edible than at an optimal experimental setting in the laboratory. Experiments with Ery4 laccase revealed that AfB1 was eliminated completely in vitro and by 26% in corn flour after treatment. There are many in vitro studies of the enzymatic degradation of aflatoxin, zearalenone, trichothecenes, and fumonisin; however, only a few are reported to be performed on food matrices, and Loi et al. claim that none of them dealt with the evaluation of the characteristics of the food matrix after the treatment. It must be ensured for the application in food and feed that there is no alteration of the matrix (protein content and quality, antioxidant activity, technological properties, etc.) when using any mycotoxin-degrading method. Therefore, the evaluation of enzymatic degradation must be assessed [76].

A very different concept to decrease the effect of aflatoxins is the vaccination of dairy animals. This could help to maintain, especially after chronic exposure, the health of animals as well as to reduce the carry-over of AfM1 to milk. Animal-borne antibodies could specifically block initial adsorption or bioactivation, toxicity, excretion in milk, or accumulation of toxins in meat by neutralization (immune-interception). Besides cows, immunization of rodents and chickens can be found in the literature. Lower mortality and reduced acute toxic effects were described for rats and rabbits after one dose of AfB1 vaccine. Although hindering of aflatoxicosis and carry-over were described, this technique is not mature and needs further investigation [7].

There are many different approaches to tackling the problem of the mycotoxin contamination of food and feedstuff. Some of them are very complicated in application, expensive, and selective in their specificity. Others do not fulfill the rules for food/feed safety. PCT offers a cost-effective, easy-to-apply, and non-toxic solution that does not need intensive storage care. Zeolites—as other aluminosilicates in general—are well-known and broadly used minerals in food and feed production, animal care, as well as human application (from food to cosmetics) for several decades now. With this present study, we offer information for two of the most health-deteriorating aflatoxins known: AfB1 and AfM1. Modern nutrition is not only oriented toward the consumption of milk products but also toward plant-based beverages. Hence, the capability of PCT to bind both toxins, while being concomitantly safe for human ingestion, offers an asset for consumers as well as for producers to ensure the consumption of non-toxic products or bind the toxins after intake before they could cause harm.

## 4. Materials and Methods

The laboratory equipment used is Good Laboratory Practice-qualified.

### 4.1. The Chemical Composition of Buffers Used in the Experiments Performed

Buffers of different kinds were used for various experiments. Table 7 provides an overview of the specific buffers, including their single components, concentrations, and pH values (according to European Pharmacopoeia [53]).

The phosphate buffer pH 6.8 contained 250 mL of 0.2 M KH_2_PO_4_ (potassium dihydrogen phosphate, 26931.263, VWR, Radnor, PA, USA) and 112 mL of 0.2 M NaOH (sodium hydroxide solution, ≥32%, extra pure, Carl Roth, Karlsruhe, Germany) in a final volume of 1000 mL.

The test solution pH 1.5 was prepared by mixing 250 mL of 0.2 M NaCl (sodium chloride, 71380, Sigma-Aldrich, St. Louis, MO, USA) with 207 mL of 0.2 M HCl (hydrochloric acid 25%, CVH Chemie-Vertrieb GmbH & Co., Hannover, Germany) and diluted with ultrapure water to a final volume of 1000 mL.

### 4.2. Artificial Digestion Fluids Preparations

Both artificial fluids were prepared according to the recommendations of the European Pharmacopoeia [53].

#### 4.2.1. Preparation of Artificial Gastric Fluid

Pepsin (P7000, Sigma-Aldrich, St. Louis, MO, USA) in the amount of 3.2 g was dissolved in 200 mL of ultrapure water (generated by Sartorius arium mini, Göttingen, Germany) prior to inactivation by autoclavation (Certoclav Tisch-Autoklav, CV-EL 12L/18L, Sterilizer GmbH, Traun, Austria) at a maximum temperature of 120 °C for 25 min (including the heating-up time). Then, the pressure inside the autoclave was reduced slowly by hand, and subsequently, the temperature of the solution was reduced to room temperature under constant stirring (Heidolph MR 3001 Heated Magnetic Stirrer, Schwabach, Germany). This deactivation step was crucial in performing ELISA testing, as previous experiments revealed that functional proteolytic pepsin degrades the antibodies provided in the ELISA kit, whereby accurate measurement was no longer possible. Afterwards, 2.0 g NaCl (sodium chloride, 71380, Sigma-Aldrich, St. Louis, MO, USA) were added and dissolved before 80 mL of HCl [1 M] (hydrochloric acid 25%, CVH Chemie-Vertrieb GmbH & Co., Hannover, Germany) were pipetted to the solution. Finally, ultrapure water (generated by Sartorius arium mini, Göttingen, Germany) was added to reach a final volume of 1000 mL. The measured pH value was 1.19 at room temperature (pH 3210i, WTW, Xylem Analytics Germany Sales GmbH & Co. KG, Weilheim, Germany).

#### 4.2.2. Preparation of Artificial Intestinal Fluid

Pancreatin (P1750, Sigma-Aldrich, St. Louis, MO, USA) in the amount of 10 g was dissolved in 200 mL ultrapure water (generated by Sartorius arium mini, Göttingen, Germany) before inactivation by autoclavation (Certoclav Tisch-Autoklav, CV-EL 12L/18L, Sterilizer GmbH, Traun, Austria) at a maximum temperature of 120 °C for 25 min (including the heating-up time). As described for the preparation of artificial gastric fluid, this step was important to ensure reliable ELISA analyses. Next, 250 mL KH_2_PO_4_ [0.2 M] were mixed with 77 mL NaOH [0.2 M] (sodium hydroxide solution, ≥32%, extra pure, Carl Roth, Karlsruhe, Germany) and added to the dissolved pancreatin solution. Then, the pH was fixed to 6.8, and ultrapure water (generated by Sartorius arium mini, Göttingen, Germany) was used to reach an end volume of 1000 mL.

### 4.3. Preparing the PCT Suspension

For preparation of the PCT (G-PUR^®^, Glock Health, Science and Research G.m.b.H., Deutsch-Wagram, Austria, Lot. 025-01-08-7-1-0) suspension, 1 g of purified clinoptilolite-tuff (PCT) was mixed vigorously with 10 mL ultrapure water (generated by Sartorius arium mini, Göttingen, Germany), and sonicated (Sonorex RK 103 H, Bandelin, Berlin, Germany) at room temperature for 15 min. Immediately prior to its use, the suspension was vortexed (Vortex Mixer SA8, Stuart, Staffordshire, UK) thoroughly again.

PCT was used in concentrations recommended for the daily intake of G-PUR (2 g), which is in the experiments performed, corresponding to 4 mg/mL in a proposed stomach volume of 500 mL.

### 4.4. Preparing the Aflatoxin B1 and M1 Stock Solutions

For preparation of the AfB1 (A6636, Sigma-Aldrich, St. Louis, MO, USA) and AfM1 (A6428, Sigma-Aldrich, St. Louis, MO, USA) stock solutions, acetonitrile (4380, Carl Roth, Karlsruhe, Germany) was used to reconstitute the lyophilized toxin. Thereafter, the solutions were aliquoted in amber glass vials and stored at −80 °C (Forma 900 Series 5905, Thermo Fisher Scientific, Marietta, OH, USA) until use.

AfB1 was used at final concentrations of up to 20 µg/L, which refers to twice the legal limit, while AfM1 was diluted to a concentration of up to 5 µg/L corresponding to the hundredfold of the legal limit for placing the product on the market.

### 4.5. Adsorption and Desorption of Aflatoxins B1 or M1 to and from Purified Clinoptilolite Tuff (PCT) in Milk and Vegetable Drinks

Aflatoxin M1 is found in the milk of grazing mammals after AfB1-contaminated food intake. During digestion, the ruminants turn the highly toxic AfB1 into the less so but still venomous AfM1 and also reduce the concentration of the latter, which is finally found in their milk. On the other hand, grain and nuts are known for their content of mycotoxins, with one of them being AfB1, which is found in the thereof produced alternative milk product. Hence, an irreversible binding and neutralization of both AfB1 and AfM1 to PCT is highly desired.

AfB1 was dissolved in phosphate buffer, pH 6.8, and testing solution, pH 1.5, to roughly determine the maximum adsorption capacity of PCT for AfB1. Both buffer and solution were chosen because their “chemical/influential effect” itself is relatively negligible, so that one could concentrate on the effect of the highly complex milk matrix (containing sugars, proteins, and fat) as a crucial factor for a proper analysis of aflatoxin binding to PCT with regard to the ELISA technique. Hence, the optimal test conditions could be determined due to this basic in vitro research. With this data gained, more complex experiments were designed, including artificial gastric and intestinal juices with both of them exhibiting a high proportion of proteins adding to the rich milk matrix. Moreover, desorption experiments were performed to determine if the binding between the aflatoxins and PCT is strong enough to pass through the digestive tract and not only of a temporary nature.

Further experiments included the examination of different cow milk products (organic and fat-reduced milk, buttermilk, whey) and organic milk from sheep and goat, as well as milk substitute products—referred to as plant-based beverages—(almond, oat, soya, hazelnut, cashew) with regard to aflatoxin decontamination by PCT.

#### 4.5.1. The Generation of an AfB1 Saturation Curve

For the characterization of the adsorption of AfB1 to PCT, AfB1 was used in various concentrations: 1 µg/L, 5 µg/L, 10 µg/L, 20 µg/L, and at two different pH values (pH 1.5 and pH 6.8). The stock solution of AfB1 was, therefore, diluted with either phosphate buffer pH 6.8 or test solution pH 1.5 to the desired concentrations.

PCT was used in a concentration of 1 mg/mL. Controls contained no PCT added.

AfB1 in several concentrations and PCT were mixed. The tests were carried out at 37 °C to imitate human body temperature and for 30 min, slowly rotating (17 rpm) to mimic the digestion process. Afterwards, the suspensions were centrifuged at 3000× *g* at room temperature for 10 min.

The AfB1 content of the single samples was analyzed by ELISA (RIDASCREEN Aflatoxin B1 30/15 ELISA kit, R1211, R-Biopharm AG, Darmstadt, Germany) according to the producer’s protocol. As declared by the producer, the detection limit (corresponding to the standard substance) refers to cereals as 1 µg/kg (ppb) and to soy as 1.7 µg/kg. The recovery rate (corresponding to the standard substance) is approximately 93% (mean recovery rate for naturally contaminated corn reference materials). The specificity corresponds to AfB1 as 100%, to AfB2 as approx. 13%, to AfG1 as approx. 29%, to AfG2 as 3.2%, and to AfM1 to approx. 1.5%.

All test approaches were performed in quadruplets per a single experiment and repeated at least two times at each pH value.

The data were visualized in the form of sorption isotherms using Excel. These isotherms were subsequently fitted to established sorption models according to the Freundlich, Langmuir, and Sips (combined Langmuir–Freundlich equation) to gain insights into the underlying sorption mechanisms. The equations were taken from Iji, Inyang, and Etuk [77].

$$\mathrm{Freundlich}\;\mathrm{equation}:\;q_{e}=K_{f}\cdot \;{c_{e}}^{1/n}$$

$$\mathrm{Langmuir}\;\mathrm{equation}:\;q_{e}=q_{max}\cdot \;\frac{K_{L}\cdot c_{e}}{1+K_{L}\cdot c_{e}}$$

$$\mathrm{Sips}\;\mathrm{equation}:\;q_{e}=q_{max}\cdot \;\frac{{\left(K\cdot c_{e}\right)}^{n}}{{1+(K\cdot c_{e})}^{n}}$$

*q_e_*…equilibrium adsorption capacity [ng/mg] or [pg/mg];*K_f_*…Freundlich constant;*c_e_*…equilibrium adsorbate concentration in the supernatant [ng/mL];
1/*n*
…heterogeneity factor [dimensionless];*q_max_*…maximum adsorption capacity [ng/mg] or [pg/mg];*K_L_*…Langmuir constant.

#### 4.5.2. Gastric and Intestinal Digestion Model of AfB1 Adsorption in Almond Drink

Almond drink was spiked with AfB1 to a final concentration of 45 µg/mL prior to further dilution with pre-warmed (37 °C) either gastric or intestinal juice to a final concentration of 10 µg/L. Then, aliquots were taken and PCT was added in concentrations of 1 mg/mL, 4 mg/mL, and 8 mg/mL to the individual samples. Aliquots without PCT served as controls. All samples were incubated rotating at 17 rpm and 37 °C for 30 min before being centrifuged at room temperature at 3500× *g* for 10 min. The resulting cream was aspirated by a water jet pump. The supernatants of the samples were immediately diluted and used afterwards for ELISA analysis.

#### 4.5.3. Comparison of AfB1 Adsorption in Different Vegetable Drinks

Milk substitutes also known as plant-based drinks (almond (Mandeldrink Joya Mandel 0% sugar; MHD 17.10.25, 03:09 E, 10 18, Mona Naturprodukte GmbH, Wien, Austria), hazelnut (Haselnussdrink Vemondo Bio Haselnuss; MHD 19.06.2025, 12:28:01, A171, GrØnvang Food ApS, Vejen, Denmark), soy (Sojadrink Joya Soja Low in sugar; MHD 20.08.25, 12:38 E, 08 22, Mona Naturprodukte GmbH, Wien, Austria), oat (Haferdrink Joya Hafer 0% sugar; MHD 28.10.25, 23:18 E, 10 30, Mona Naturprodukte GmbH, Wien, Austria), and cashew (SPAR Veggie veganer Bio-Drink Cashew, SPAR Österr. Warenhandels-AG, Salzburg, Austria)) were spiked with AfB1 to a final concentration of 1000 µg/L prior to further dilution with pre-warmed (37 °C) phosphate buffer pH 6.8 to a final concentration of 200 µg/L. Then, aliquots were taken, and PCT was added in a concentration of 1 mg/mL to the individual samples. Aliquots without PCT served as controls. All samples were incubated rotating at 17 rpm and 37 °C for 30 min before being centrifuged at room temperature at 3500× *g* for 10 min. The resulting cream was aspirated by a water jet pump. The supernatants of the samples were immediately diluted appropriately and used afterwards for ELISA analysis.

The experiments were performed at least two times in quadruplets per approach.

#### 4.5.4. The Generation of a AfM1 Saturation Curve

To characterize the adsorption of AfM1 to PCT, AfM1 was used in various concentrations: 50 ng/L, 100 ng/L, 250 ng/L, 500 ng/L, 1000 ng/L, and at two different pH values (pH 1.5 and pH 6.8). The stock solution of AfM1 was therefore diluted in cow milk to a final concentration of up to 5 µg/L. This working solution was further diluted 1:5 (*v*/*v*) with either phosphate buffer pH 6.8 or test solution pH 1.5 to the desired concentrations.

PCT was used in a concentration of 4 mg/mL. Controls contained no added PCT.

AfM1 in several concentrations and PCT were mixed. The tests were carried out at 37 °C to imitate human body temperature and for 60 min, slowly rotating (17 rpm) to mimic the digestion process.

Afterwards, the suspensions were centrifuged (MEGA STAR 1.6R, VWR, Radnor, PA, USA) at 3500× *g* at 10 °C for 10 min. Then, the resulting cream was aspirated by a water jet pump. The aqueous phase was further analyzed.

The free AfM1 of the single samples was quantified by ELISA (RIDASCREEN Aflatoxin M1, R1121, R-Biopharm AG, Germany) according to the producer’s protocol. As declared by the producer, the detection limit (corresponding to the standard substance) refers to milk as 5 ng/L (ppt). The recovery rate (corresponding to the standard substance) for milk is 100% (in spiked samples). The specificity corresponds to AfM1 as 100%, and to AfM2 to <10%.

All test approaches were performed in quadruplets per a single experiment and repeated at least three times at each pH value.

#### 4.5.5. The Kinetics of AfM1 Adsorption to PCT at pH 1.5 and pH 6.8

To characterize the binding of AfM1 to PCT in a time-dependent manner, the AfM1 ELISA was used. Cow milk was contaminated with AfM1 and diluted 1:5 with test solution pH 1.5 or phosphate buffer pH 6.8 to a final concentration of 500 ng/L. The phosphate buffer pH 6.8 and the test solution pH 1.5 were preheated in a water bath (Medingen WB 10, P-D Industriegesellschaft mbH Prüfgerätewerk Dresden, Dresden, Germany) to human body temperature (37 °C) before use. Purified clinoptilolite-tuff was diluted in the same buffer to gain an end-concentration of 4 mg/mL. Controls containing AfM1 without PCT in suspension and samples with added AfM1 and PCT were incubated at 37 °C under rotation (17 rpm, rotator SB3, Stuart, Staffordshire, UK) for time periods of 1 min, 5 min, 10 min, 20 min, and 30 min. The samples were centrifuged (MEGA STAR 1.6R, VWR, Radnor, PA, USA) at 3500× *g* for 10 min at 10 °C. The resulting cream was aspirated by a water jet pump prior to dilution in sample buffer (contained in the ELISA kit), and the content of free AfM1 in the supernatants was determined via ELISA, following the manufacturer’s instructions. Measurements were performed at a wavelength of λ = 450 nm by use of a plate reader (Biotek synergy HT, BioTek Instruments, Inc., Winooski, VT, USA) equipped with Gen5 Secure 3.03 software (BioTek Instruments). All test approaches were performed in quadruplets.

#### 4.5.6. The Creation of a Simple Digestion Model with Incremental pH for AfM1

The digestion process itself is a continuous process. Although in vitro research is regimented to rudimentary models comparatively to in vivo experiments, the effort towards a more life-mimicking test setting is of fundamental interest. In the following, the binding of AfM1 to PCT in the stomach milieu (test solution pH 1.5) prior to stepwise increase in pH (by addition of sodium acetate and TRIS) to intestinal level (pH 7.2) was performed.

Cow milk was spiked with AfM1 (300 ng/L) and diluted with prewarmed (37 °C) testing solution pH 1.5 to a final concentration of approximately 60 ng/L to be above the legal European limit of 50 ng/L. Then, the samples containing PCT (final concentration 4 mg/mL) and controls without PCT were incubated for 30 min, gently shaking at 30 rpm (Sea Star Digital Orbital Shaker, Heathrow Scientific LLC, Vernon Hills, IL, USA) and 37 °C for 30 min. Afterwards, aliquots were taken from each approach for analyses prior to the addition of sodium acetate (106268, anhydrous for analysis, Sigma-Aldrich, St. Louis, MO, USA) and TRIS (Tris-(hydroxymethyl)aminomethane, P33621, VWR International GmbH, Darmstadt, Germany) to raise the pH of each sample. The samples were kept under the same conditions as before for another 30 min. Again, an aliquot was taken from each sample, and subsequently sodium acetate and TRIS were added again. The experiment was continued by another incubation at 37 °C for 30 min. Once more, aliquots of the remaining quantities of samples and controls were taken out. This was followed by a final incubation for another 150 min prior to taking final aliquots.

All of the aliquots were immediately centrifuged after drawing at 10 °C and 3500× *g* for 10 min. The resulting cream was aspirated by a water jet pump. Afterwards, the pH value of each aliquot was defined by both pH test strips (Dosatest pH 0–14 indicator test strips, 85410.601, VWR International GmbH, Darmstadt, Germany) and by a pH meter (pH 3210i, WTW, Xylem Analytics Germany Sales GmbH & Co. KG, Weilheim, Germany).

The content of free AfM1 was analyzed promptly by ELISA measurement. All test approaches were performed in quadruplets.

#### 4.5.7. PCT-Adsorption of AfM1 by Mimicking In Vivo Conditions Using Artificial Gastric and Intestinal Fluids

In this experimental setup with artificial gastric fluid or intestinal fluid, various final concentrations of PCT (ranging from 1 mg/mL, 4 mg/mL, 8 mg/mL, and 16 mg/mL, to 32 mg/mL) were applied. AfM1 spiked cow milk was diluted with either artificial gastric or intestinal fluid to a final concentration of 60 ng/L. Samples without PCT served as controls. The incubation lasted 60 min at 37 °C, rotating at 17 rpm. After that, the samples were centrifuged at 10 °C and 3500× *g* for 10 min. The resulting cream was aspirated by a water jet pump. Then, the samples were diluted and analyzed directly by AfM1 ELISA according to the manufacturer’s recommendations. All test approaches were performed in quadruplets.

#### 4.5.8. Comparative Analysis of AfM1 Sorption by PCT in Milk and Milk Products of Cow, Goat, and Sheep

The universality of PCT towards binding AfM1 was analyzed using different cow milks (conventional—Frische Vollmilch (Milsani ST1/2 18:27/G, MHD 14.10., Gmundner Molkerei, Gmunden, Austria), organic—Bio Kuhmilch (Ja Natürlich! L1272 16:11, MHD 23.10.24, NÖM, Baden, Austria), fat reduced—Leichte Milch (Milsani ST1/3 17:50/E, MHD 14.10., Gmundner Molkerei, Gmunden, Austria)) and milk products (whey—Molke (Latella, L7.2/218/19:13 A/S, MHD 09.11.24, Berglandmilch, Wels, Austria), buttermilk—Buttermilch (Clever 1527404:43, MHD 23.10.2024, NÖM, Baden, Austria)) as well as organic milk derived from sheep (Bio Schafmilch (Ja Natürlich! MHD 27.10.24, 01444, NÖM, Baden, Austria)) and goat (Bio Ziegenmilch (Ja Natürlich! MHD 20.10.2024, 08:13, NÖM, Baden, Austria)).

All milk and milk products were spiked with AfM1 to a concentration of 500 ng/L corresponding to ten times the European legal limit for license to market prior to dilution with phosphate buffer pH 6.8 to a final concentration of 100 ng/L. This buffer-toxin solution was pre-warmed to 37 °C in a water bath before mixing aliquots with PCT in a final concentration of 4 mg/mL. Aliquots without PCT served as controls. All of the milk and milk-derived products approaches (with and without PCT) were performed in quadruplets.

The samples were incubated rotating (17 rpm) at 37 °C for 30 min. Afterwards, the samples were centrifuged at 3500× *g* at 10 °C for 10 min. The resulting cream was aspirated by a water jet pump. Then, the samples were diluted and analyzed directly by AfM1 ELISA according to the manufacturer’s recommendations.

### 4.6. Determination of Aflatoxin AfB1 and AfM1 Content via ELISA

#### 4.6.1. Analysis of AFB1 Content by ELISA

The content of AfB1 in the supernatants was determined by RIDASCREEN Aflatoxin B1 30/15 ELISA kit (R1211, R-Biopharm AG, Germany) according to the manufacturer’s advice before measurement of the samples in a spectrophotometer at a wavelength of λ = 450 nm. The absorbance is inversely proportional to the AfB1 concentration in the sample, because this is a competitive enzyme immunoassay for the quantitative analysis of aflatoxin B1 in cereals and feed. All required reagents are contained in the test kit. It has an AfB1 limit of detection according to 0.9 µg/L in oats, 1.6 µg/L in soy, 0.7 µg/L in almonds, 0.9 mg/L in cashew, and 0.5 µg/L in hazelnuts. The mean recovery rate refers to 100% in almonds and 88% in hazelnuts. The specificity for AfB1 is 100%, those for AfB2, AfG1, AfG2, and AfM1 are approximately 13%, 29%, 3.2%, and 1.5%, respectively. Standards are provided in a range corresponding to 0 µg/L, 1 µg/L, 5 µg/L, 10 µg/L, 20 µg/L, and 50 µg/L.

#### 4.6.2. Analysis of AFM1 Content by ELISA

AfM1 was detected by using the RIDASCREEN Aflatoxin M1 ELISA kit (R1121, R-Biopharm AG, Germany), which is a competitive enzyme immunoassay for the quantitative analysis of aflatoxin M1 in milk and milk powder. All required reagents are contained in the test kit. It has an AfM1 limit of detection of 5 ng/L (ppt) in milk and a recovery rate referring to 100% in spiked milk samples. The specificity according to AfM1 is 100% and to AfM2 is <10%. AfM1 Standards are provided for 0 ng/L, 5 ng/L, 10 ng/L, 20 ng/L, 40 ng/L, and 80 ng/L.

The samples were measured in a spectrophotometer at a wavelength of λ = 450 nm. The absorption is inversely proportional to the AfM1 concentration in the sample.

## 5. Conclusions

Aflatoxins are among the most highly toxic fungal compounds for humans and higher animals. In particular, AfB1 has a strong venomous action, which is found to a slightly lesser extent in its metabolite AfM1. Oral and dermal contact, as well as inhaling, can lead to severe health problems. Since the primary route of aflatoxin exposure is through contaminated food and feed, preventing their incorporation into the diet is essential to reduce the risk of uptake into the body. Although a broad spectrum of chemical, physical, and biological techniques is applied to avoid mold colonization and synthesis of the toxins towards the mitigation of their action when already present, a complete eradication is impossible. Hence, a cost-effective capture of aflatoxins from food and feed in the digestive tract is beneficial to impede acute and chronic aflatoxicosis.

Since ancient times, humans, like animals, have consumed minerals to improve or maintain their physical condition. Here, a purified clinoptilolite-tuff (PCT) was applied in in vitro experiments to bind both AfB1 and AfM1 from several plant-based beverages, as well as from different dairy products deriving from various species. Moreover, artificial digestion experiments with enzymes and changing pH values, as well as temperature, and adjusted incubation times mimicking a living organism were implied to gain a more lifelike impression of a possible action towards the sorption of toxin to PCT, as simple binding experiments in buffer or other solutions grant optimal conditions for the reaction without any potential spoilers. Also, desorption tests were performed to analyze the durability of the detoxification.

Almond, hazelnut, soy, oat, and cashew drinks could be cleared from artificial, unrealistic high AfB1 contamination efficiently (no European legal limit set). AfM1 contaminated whole, fat-reduced, buttermilk, and whey from cows, as well as organic milk from sheep and goat, were spiked with 10 times the legal European limit. A daily dose recommended for humans of PCT could neutralize the toxin to a level beyond the legal limitation. Desorption analyzes revealed no significant amount of free AfM1 was available after 4 h of incubation. Both toxins bound with high affinity and velocity in a dose-dependent manner to PCT.

## Figures and Tables

**Table 1:** Examples of plant-based beverages are classified into different subgroups. Plant-based beverages may be composed of only a single plant or be a mixture of two or more herbal ingredients [16,17,18,19].

Origin	Examples
Poaceae	rice, oat, corn, spelt, teff
Fabaceae	soy, lupin, peanut, pea, adzuki bean, cowpea
Amaranthaceae	amaranth, quinoa
Anacardiaceae	cashew, pistachio
Pedaliaceae	sesame
Arecaceae	coconut
Betulaceae	hazelnut
Juglandaceae	walnut
Rosaceae	almond, apricot seed
Cannabaceae	hemp
Linaceae	flax
Asteraceae	sunflower

**Table 2:** Examples of physical, chemical, and biological ways to mitigate aflatoxins in food and feed [4,5,7].

Method	Examples
physical	sorting, dehulling, steeping, wet or dry milling, heat treatment, irradiation (gamma, solar, UV, microwave, near-infrared), pulsed electric fields, carbon filtration, high-pressure cooking, ultrasound, plasma-treatment, electrolyzed water, pulsed light
chemical	adsorbents (e.g., clays), acids (organic and inorganic), bases (ammoniation), enzymes (redox-active), gases (e.g., ozonation, chlorine dioxide)
biological	bacteria (e.g., lactic and non-lactic acid), microfungi (e.g., yeasts), genetic engineering (e.g., generation of *A. flavus* lacking aflatoxin-producing ability, host-induced gene silencing), vaccination

**Table 3:** Basic characteristics of PCT used in these experiments (analytical methods according to Haemmerle et al. 2021 [47]).

**Minerals**	
Main mineral	clinoptilolite (>80 wt%)
Minor minerals	cristobalite, albite, orthoclase, quartz
Trace minerals	biotite, anorthite
**Particle size distribution**	
D(0.5) [µm]	3.1 ± 0.5
**Cation exchange capacity**	
[mol/kg]	>0.75

**Table 4:** Comparison of AfB1 sorption by PCT with regard to different types of plant-based beverages. Mean values of 2 individual experiments in quadruplets each are given.

Type of Plant Drink	Initial pH Value	Initial AfB1 Concentration [µg/L]	AfB1 Concentration After PCT Incubation [µg/L]	AfB1 Sorbed by PCT [%]	Free Residual AfB1 [%]
Almond	7.83	213.2	49.7	76.7	23.3
Hazelnut	7.03	219.8	47.9	78.2	21.8
Soy	7.74	227.4	59.2	74.0	26.0
Oat	7.27	206.7	43.0	79.2	20.8
Cashew	6.96	216.7	47.0	78.3	21.7

**Table 5:** pH values of aliquots from controls and PCT-containing samples during incubation. Aliquots of quadruplets were pooled prior to measurement, except those after 240 min incubation time—here each aliquot was tested by pH meter as it was the longest time of probable PCT influence on pH value. Tests with pH strips were always performed only with pooled aliquots, as they are not sensitive enough to define marginal differences. One representative experiment is shown.

Aliquotes	Incubation Time [min]	pH Indicator Test Strips	pH Meter
control	30	2.0	2.33
PCT	30	2.0	2.39
control	60	5.5	5.48
PCT	60	5.5	5.55
control	90	8.0	7.86
PCT	90	8.0	7.81
control	240	8.0	7.80, 7.87, 7.88, 7.87
PCT	240	8.0	7.83, 7.77, 7.83, 7.82

**Table 6:** Content of fat and protein as declared by the single manufacturer in various milk and milk products tested.

Animal	Type of Milk Product	Fat Content [%]	Protein Content [g]
Cow	Buttermilk	1	3.2
Cow	Whey	<0.5	0.6
Cow	Fat-reduced milk	0.9	3.4
Cow	Milk (conventional)	3.5	3.3
Cow	Organic milk	3.5	3.4
Sheep	Organic milk	4.5	4.6
Goat	Organic milk	2.8	3.1

**Table 7:** Preparation of phosphate buffer, pH 6.8, and test solution, pH 1.5. Both phosphate buffer and test solution were prepared from chemicals with the highest purification grade. Ultrapure water (generated by Sartorius arium mini, Göttingen, Germany) was used as solvent. pH values were fixed by the addition of either 0.1 M NaOH solution or 0.1 M HCl solution. pH measurement was performed by use of a precision pH meter (pH 3210i, WTW, Xylem Analytics Germany Sales GmbH & Co. KG, Weilheim, Germany) in combination with a suitable pH electrode (Sentix 81, WTW, Xylem Analytics Germany Sales GmbH & Co. KG, Weilheim, Germany) featuring an integrated NTC temperature sensor at room temperature.

**Phosphate Buffer** **[pH]**	**KH_2_PO_4_ Solution** **[0.2 M]** **Volume [mL]**	**NaOH Solution** **[0.2 M]** **Volume [mL]**	**Final** **Volume** **[mL]**
6.8	250	112	1000
**Test Solution** **[pH]**	**NaCl Solution** **[0.2 M]** **Volume [mL]**	**HCl Solution** **[0.2 M]** **Volume [mL]**	**Final** **Volume** **[mL]**
1.5	250	207	1000

## Data Availability

The original contributions presented in this study are included in the article. Further inquiries can be directed to the corresponding authors.
